# AutoCellSeg: robust automatic colony forming unit (CFU)/cell analysis using adaptive image segmentation and easy-to-use post-editing techniques

**DOI:** 10.1038/s41598-018-24916-9

**Published:** 2018-05-08

**Authors:** Arif ul Maula Khan, Angelo Torelli, Ivo Wolf, Norbert Gretz

**Affiliations:** 1 Medical Faculty Mannheim, Medical Research Center, University of Heidelberg, Mannheim, 68167 Germany; 2 Mannheim University of Applied Sciences, 68163 Mannheim, Germany

## Abstract

In biological assays, automated cell/colony segmentation and counting is imperative owing to huge image sets. Problems occurring due to drifting image acquisition conditions, background noise and high variation in colony features in experiments demand a user-friendly, adaptive and robust image processing/analysis method. We present AutoCellSeg (based on MATLAB) that implements a supervised automatic and robust image segmentation method. AutoCellSeg utilizes multi-thresholding aided by a feedback-based watershed algorithm taking segmentation plausibility criteria into account. It is usable in different operation modes and intuitively enables the user to select object features interactively for supervised image segmentation method. It allows the user to correct results with a graphical interface. This publicly available tool outperforms tools like OpenCFU and CellProfiler in terms of accuracy and provides many additional useful features for end-users.

## Introduction

Biologists perform numerous experiments in the laboratory based on cell culture under different conditions and acquire images for analysis. However, this leads to a huge amount of image data containing many cells/colonies depending upon the type of experiment being performed and the effects under observation.

The colony forming units (CFUs) or cells found in images produced during microbiological assays are hard to analyze manually in an efficient way, especially when the images and/or the amount of CFUs/cells are large in number. One may be able to count the CFUs manually provided that adequate time is available. However, to make a quantitative analysis based on other CFU features is a nontrivial and even more complex task than just enumerating all the colonies. For instance, in some experiments, it is desirable to see the change in cell/CFU morphology or size by the effects induced in the cells/CFUs under control and test conditions. When the difference in change to be observed between the experiments is marginal, finding the size and number of CFUs/cells with great accuracy becomes imperative. Therefore, an automatic, high accuracy analysis is needed.

In order to automate the analysis, one may need to develop an algorithm that automatically segments the colonies from the unwanted background in such a way that CFU boundaries are obtained. Based on this segmentation, not only the counting of CFUs should be performed, but additional features of interest such as size, shape etc. should also be extracted. However, this leads to individual steps of a typical image analysis pipeline: Noise removal, image segmentation, feature extraction, feature selection, object counting/classification. For each individual step, algorithmic parameters are required to obtain the targeted colonies in each individual image. For the analysis of the whole data set, more flexible parameters must be chosen, since the individual images may vary greatly from one another in object size, object morphology and image intensity. From a user’s stand point, the parameters should be intuitive such as setting the minimum and maximum size of colonies expected in an assay. Therefore, an abstract a priori knowledge by the user about the size, number, shape and other features of the colonies to be found should be defined. Against the backdrop of analyzing the whole data set, supervised automatic image analysis could be implemented with intuitive and flexible parameters set by users.

Some of the major problems hindering a good CFU analysis are high CFU density, imperfection and impurities in suspension medium, inherent background acquisition noise, inconsistent illumination artifacts, luminous reflectance and other visible artifacts on the containers boundary, close proximity between CFUs or with container boundaries and low image resolution.

Automated colony segmentation and counting has long been a topic of interest in microbiology^1^. Different cell segmentation methods have been used^2–7^. For cell colonies, various solutions exist as software tools as summarized in^8^ for analyzing CFUs. There are few commercial tools such as ColonyDoc-ItTM by UVP (Analytik Jena AG) (*UVP*), STEMvisionTM by STEMCELL Technologies (*STEMCELL*) etc. These programs are expensive and have undisclosed algorithms running during graphical user interface (GUI) operations.

Although MATLAB is a commercial software, programs based on it are quite popular as they are more intuitively programmable and can also be deployed on all major operating systems (Windows/Mac/Linux) using the freely available MATLAB run time compiler. Some MATLAB-based CFU counting solutions are CHiTA^9^ and NICE^10^. CHiTA is based on circular Hough transform making it liable to discard more eccentric segments. NICE employs a combination of extended minima and thresholds but is not suited for a variety of CFUs differing in size and shape from the ones given in their sample data. These software solutions are also not able to detect the container boundaries containing the CFUs and consider the whole image as the searching area for CFUs. Additional cropping potentially improves the outcome but nevertheless, many erroneous segments are detected. Another MATLAB-based software^11^, developed to work in conjunction with a special hardware, provides no high throughput solution for input images. The method is based on adaptive thresholding combined with global thresholding. However, with almost no parametric selection freedom, it is bound to perform worse for other data sets.

General preference for freely available open-source tools is evident in the near future. Many such tools exist that facilitate the user to write their own tailor-made programs. For instance, a very popular choice recently is OpenCFU^12^ written in C++ that uses open source computer vision library OpenCV (under BSD license). The standalone application is available for both operating systems Linux and Windows, with compilation instructions for GNU/Linux only (*OPENCFU* last checked: March 13, 2018). The advantage of using OpenCFU is that it is fast and works on a variety of images with simple parameter settings. It is quite good at recognizing very few false positives. However, it is prone to underestimate the number and the size of colonies due to its strict CFU selection criteria since it aims for more circular objects.

CellProfiler (*CellProfiler*) is another popular open-source software^13^. Recently in^8^, a custom-made pipeline was constructed in CellProfiler to detect, count and quantify CFUs. Another commonly used platform is ImageJ^14^ that is Java-based and has its own GUI. Cai *et al*. provided a macro that works with ImageJ to analyze CFUs^15^ commonly called IJM. In^8^, another macro based on edge detection algorithm called Cell Colony Edge was developed in ImageJ to adapt to particular images. The main problem in using custom made pipelines and macros is that they may not perform well on different images and user always have to do some modifications not only in the parameter values but also in number and order of steps involved.

More recently, machine learning based approaches became popular^16^. Ilastik^17^ is a beginner-friendly framework that uses a Random Forest classifier^18^ to classify image pixels into user-defined classes based on the interactive graphical pixel labeling. It offers 37 predefined features that a user can select for the classification process. In^19^, a specific solution for bacteria colony counting based on convolution neural networks was proposed. It requires completely labeled data with ground truth for testing and evaluation. It classifies colony conglomerations into classes of one to six colonies per aggregation and rejects false positives using an outliers class. However, it does not extract and compare colony features and has no GUI for experienced users. In a recent work^20^, a user-friendly software called fastER is proposed for fast and robust cell segmentation. It uses extremal regions and consequent scoring applying support vector machine (SVM) with Gaussian kernel. It is programmed in C++ and a standalone GUI is freely available for use. The main problem with fastER is that it tends to yield a lot of false positives when there is a colony container visible in the image data. Apart from that, it does neither provide any kind of freedom in parameter selection nor does it allow the user to correct the results obtained.

The problem of generality is cumbersome, as not everybody has the will, expertise and/or time to reprogram the existing solutions. Moreover, the parameter selection process also demands a lot of experience of the user. Many popular software choices suffer from the lack of quick and intuitive parameter selection. There is definitely a need for more user-friendly software that is appealing to programmers, advanced users and the beginners/new users alike.

Furthermore, artifacts in images such as CFU container intensity results in false positives. Automatic dish extraction is rarely used^21,22^. In these papers, a contrast limited adaptive histogram equalization in addition to morphological filters is applied, which often leads to false positives if suitable post-filtering is not employed. Semi automated methods also exist e.g. 3-points selection^23^ and using shapes for cropping in CellProfiler. Additionally, unknown acquisition conditions and object locations can adversely affect the results even with tuned parameters. A correction step is, therefore, imperative to be integrated in any image analysis pipeline. This step should be intuitive, interactive, flexible and fast-running in performance.

**Figure 1:**
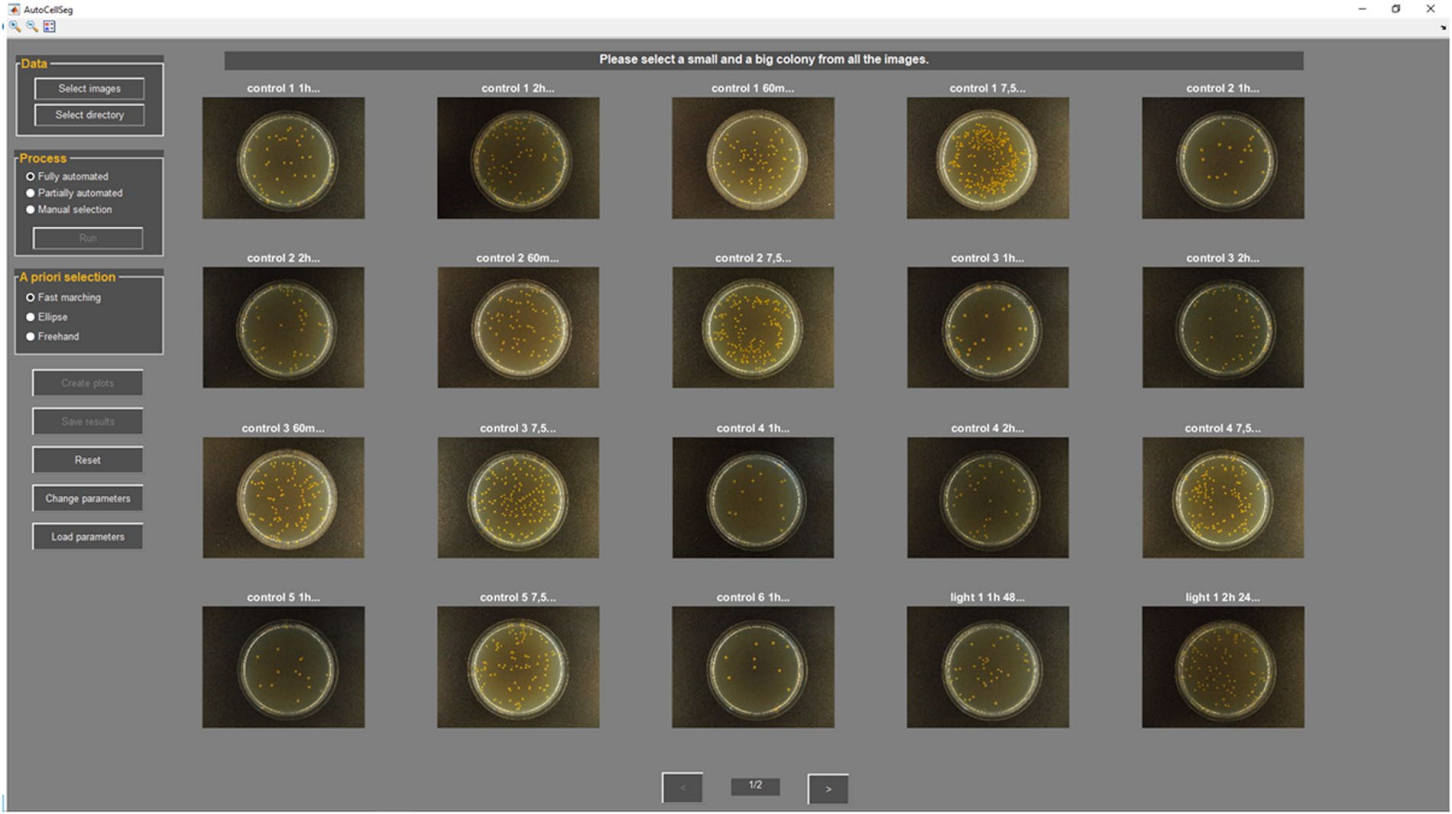
The interactive window of the AutoCellSeg GUI after loading the image data.

One solution could be to use feedback-based segmentation mechanisms using supervision by intuitive and interactive selection of a priori features for objects to be found in an image. In addition, a comparison in cell/CFU count and size without the need of transferring the results to a different tool is often desired. Here we present AutoCellSeg (Fig. 1), a new MATLAB-based tool, that contains interactive and easy-to-use features selection, post-editing and analysis.

## Results

In this chapter, results from the new benchmark data we created and other data sets used in previous works are presented. AutoCellSeg was compared to other software tools using various quality measures.

### New benchmark data

We labeled 12 images, which were acquired at our laboratories, for ground truth from different bacterial species (3 images each) including *E.coli*, *Klebsiella pneumoniae*, *Pseudomonas aeruginosa* and *Staphylococcus aureus*. The images were labeled by delineating boundaries of colonies using Adobe Photoshop and then with MATLAB to create labeled binary images. Consequently these ground truth images are used to extract: (1) colony count (2) size of each individual colony in pixels.

Previous reports on the specificity and sensitivity of tools used for colony counting are predominantly based on the total count only. Such a comparison gives a lesser insight into the plausibility of colonies especially in terms of the size detected. The size of each colony could be of equal importance in many experiments. Therefore, a quality criterion based on segmentation precision should also be incorporated. The quality measure *Q*, based on^24^, was used to evaluate the outcome of the segmentation process (see Supplementary Data 1 for more details). *Q* takes into account: difference in segment count with respect to ground truth (*q*_1_), and number of misclassified segment pixels (*q*_2_).

**Figure 2:**
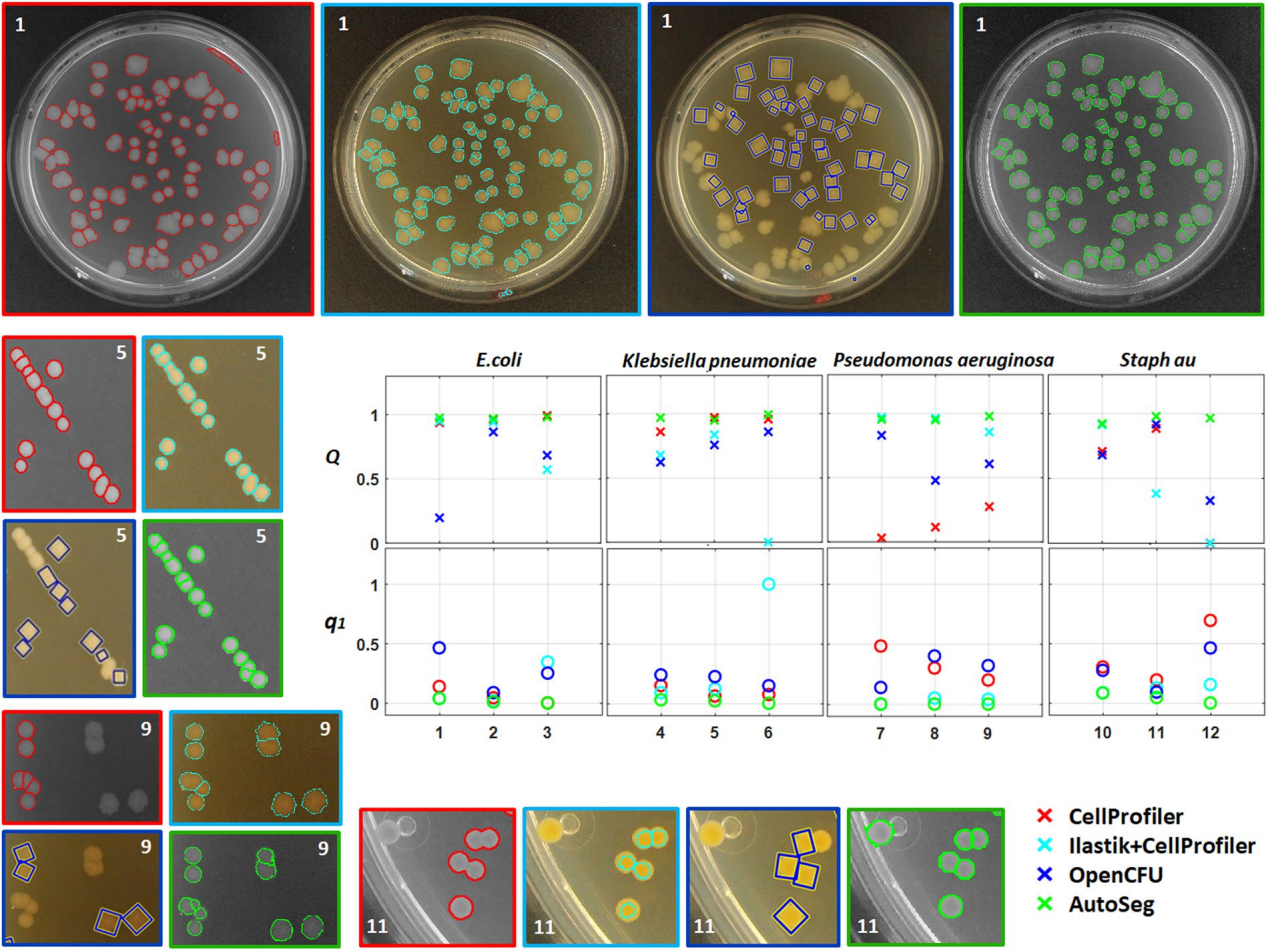
Results of the methods used on the new benchmark: CellProfiler (red), Ilastik + CellProfiler (cyan), OpenCFU (blue) and AutoCellSeg (green). One image from each species is selected (indicated by the image number on the image corners) to demonstrate the comparison. The graphs show segmentation/counting (*Q* on y-axis of first row) and deviation from manual count (*q*_1_ on y-axis of second row) for all methods used based on the complete benchmark (on x-axis). *Q* incorporates the measures for both size and count of CFUs.

Using *Q* on new benchmark, different tools were compared (see Fig. 2). Since both IJM and ImageJ Edge fails in the presence of CFU container in the image, we opted for alternate solutions like OpenCFU. We also developed a custom-made pipeline in CellProfiler, based on the work done by^8^, and a combination of Ilastik and CellProfiler for comparison with AutoCellSeg. While AutoCellSeg extracts the Petri dish area automatically, CellProfiler and OpenCFU do not. In OpenCFU a region of interest (ROI) in form of a 3-point circle or complex polygon is needed in order to extract the information only present inside the container. The “Auto-Petri” function mentioned in^12^ is nowhere to be seen on its GUI and neither it runs perfectly at the back-end since in several results noise outside the dish was segmented. A separate pipeline was used for each species and can be found in the AutoCellSeg repository: https://github.com/AngeloTorelli/AutoCellSeg/tree/master/DATA/Benchmark. For instance, the CellProfiler pipeline and results for *E.coli* can be found under its own folder: https://github.com/AngeloTorelli/AutoCellSeg/tree/master/DATA/Benchmark/E.coli/CellProfiler. Due to manual tuning of parameters and selecting the perti dish manually, CellProfiler may not be the solution for high throughput and fully automated segmentation. The segmentation outcome produced by CellProfiler is good for benchmark data sets, it was hence included for the comparison.

The inclusion of Ilastik was done because it delivers reasonable pixelwise prediction by using supervised machine learning. For each bacterial specie, only one image was taken for labeling pixels of the background and colonies (two class problem) using all available features until the resulting segmentation was close to the ground truth. The segmentation results were exported as tiff files and thereafter combined with CellProfiler pipeline for quantification.

OpenCFU avoids false detections owing to its careful colony selection criteria. In OpenCFU the user has to be careful about the range of colony size to be input manually using the slider bar. If a very small lower radius is selected, OpenCFU produces a lot of false positives in addition to detecting the noise both inside and outside CFU container. Conversely, if a lower radius is set to a suitable value (i.e. 25–35 in case of *Staphylococcus aureus*), the colony count is largely underestimated. A user first has to try different values for lower radius in order to get a good segmentation result. The selection of these values may be trivial to the expert user, but even then it still requires trial and error if different data sets with varying resolutions are to be evaluated. This problem is adequately rectified in AutoCellSeg, where the user just has to click on a small and a big colony as the lower and higher radius are then extracted automatically using fast marching method.

The other main issue in OpenCFU is that there is no fully automated batch throughput solution. A user has to manually click on the ‘>’ tab on the GUI to process the next image. This hinders its ability to be used for larger data sets even when global parameters for segmentation/counting could be safely set. Conversely, AutoCellSeg allows to be used in a fully automated mode without any kind of human intervention during its running process.

Both OpenCFU and AutoCellSeg were operated in semi-automatic mode (see ‘Process Selection’ in Supplementary Data 2 for different modes of operation). The results of segmentation were obtained as binary masks directly from AutoCellSeg and CellProfiler pipeline. However, in the case of OpenCFU, usable features for required quality comparison are center and radius of the colonies. This is another drawback of OpenCFU, that one cannot obtain a binary mask directly for comparison of count/size of colonies with ground truth. Using center and radius of colonies, we reconstructed binary circles to emulate the colonies and used watershed segmentation to separate the overlapping colonies. This was done to match the number and size of colonies as extracted from OpenCFU software. All three binary masks obtained were compared against ground truth for the total count and size of colonies using *Q*.

Some example results from the new benchmark image data set are shown in Fig. 2. The red, cyan, blue and green colors are used to represent CellProfiler, Ilastik + CellProfiler, OpenCFU and AutoCellSeg respectively. In some cases, CellProfiler delivers very good segmentation. The results of Ilastik with CellProfiler in batch operation was not drastically better than OpenCFU or CellProfiler. Nevertheless, the combination of the two produces comparable results to AutoCellSeg when labeling all images individually but it comes at the expense of both time and labeling effort. AutoCellSeg still outperforms the other solutions in terms of the quality *Q* of the segmentation outcome. The individual comparison can be seen in the graphs of Fig. 2. The overall comparison between AutoCellSeg, OpenCFU and CellProfiler and the combination of Ilastik + Cellprofiler is given in Table 1 using an average *Q* value (*Q*_*m*_) and average *q*_1_ value (*q*_1,*m*_).

**Table 1:** Quality comparison of different methods using benchmark images.

Software	CFU container extraction	Post-processing	Benchmark evaluation *Q*_*m*_	Deviation in count *q*_1,*m*_ (%)
AutoCellSeg	Automatic	Addition + removal	0.97	1.9
OpenCFU	Manual (ROI mask)	Removal	0.65	25.8
CellProfiler	Manual	N/A	0.64	22
Ilastik + CellProfiler	N/A	N/A	0.67	17.2

The *Q*_*m*_ and *q*_1,m_ values shown in the last two columns are the average values for all 12 images used.

### Control/ test analysis

Unlike other CFU analysis software solutions, AutoCellSeg has the possibility to compare different modes of data. For example, in a microbiological experiment, one may need to know the changes in CFU morphology and number that occur during treatment. The test was to irradiate the colonies with blue light to observe the differences in size and count of colonies. Therefore, AutoCellSeg prompts the user to select control and test images in order to make an experiment-wise comparison. For example, we chose a set of control and test images from *E*. *coli*. A test (i.e light irradiation) is performed to observe the CFU size change with respect to control images.

**Figure 3:**
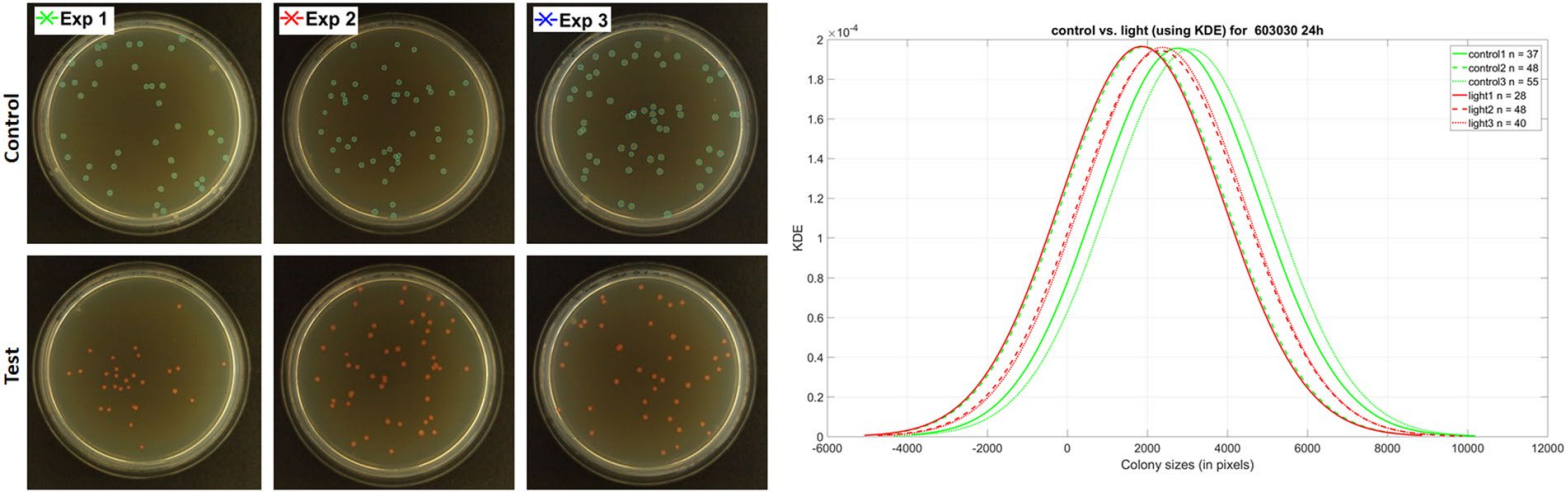
Analysis of control/test images: First and second row on left show the segmentation results for control (light blue) and test (red) images respectively. On the right side, Kernel Density Estimation (KDE) plot is displayed on y-axis for colony sizes in pixels (x-axis). The bandwidth (*bw* = 2000) here determines the smoothness in the curves. Green lines in plot represent control experiment results, where as red lines represent test experiment (light irradiation in our case) results. The number of colonies found in each image is denoted by *n* using red/green color based on the experiment type (light/control).

There are three test/control pairs represented by numbers. The images are loaded in AutoCellSeg and the parameters are adjusted as shown in Supplementary Data 3. The program is then run in the semi-automatic mode. The correction is done at each individual image and when all images are processed, the user can choose to display comparison plots. The segment outlines (the output after running the process) are the results of AutoCellSeg detection. The segmentation results for all the images are shown in: https://github.com/AngeloTorelli/AutoCellSeg/tree/master/DATA/control_test. Cyan outlines represent control CFU delineation and red represents the outlines from the test experiment depending upon the naming convention. For each pair of data set, a Kernel Density Estimation (KDE) function is plotted depicting the size distribution of colonies (in pixels) as shown in Fig. 3. The function returns a probability density estimate of CFU size. The estimate is based on a normal kernel function, and is evaluated at equally-spaced points, *x*_*i*_, that cover the range of the data in *x*. Here, *x* is used to describe the CFU size *a*.

In the graph of Fig. 3, the green curve depicts the size distribution in control colonies and red dashed curve shows the size distribution of CFUs after light irradiation. The smoothness of the curve is controlled by a bandwidth parameter *bw*. In this data set, we used *bw* = 2000. Using smaller *bw* values could result in less smoother curves and probably more than one peaks depending upon the variance in size of CFUs. The negative values at the start of x-axis are due to the extrapolation of smoothing function on the left side of the peak in x-data. This does not indicate the existence of colonies with a negative CFU area.

**Figure 4:**
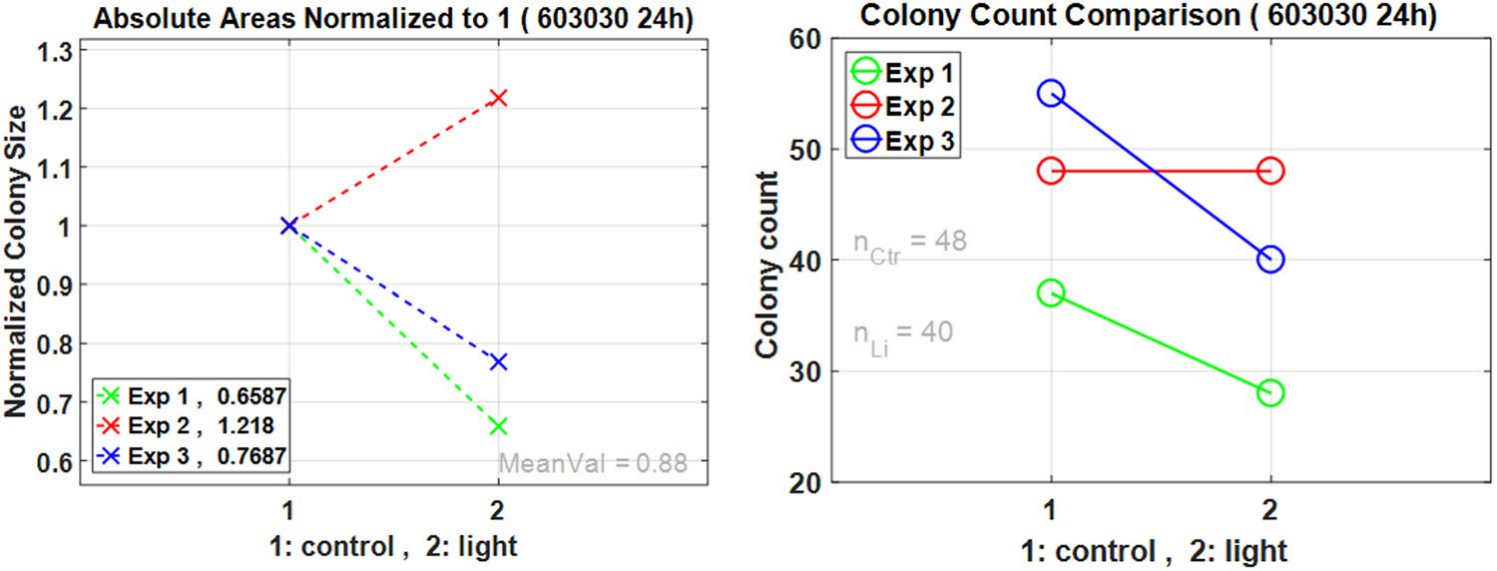
Overview plots of change in total count and size of CFUs after test experiments. The plot on left shows the change in absolute colony sizes (normalized to 1 for each individual control/test pair). This is shown to express the change in size for each pair of experiments. The mean value of the three experiments is given as MeanVal = 0.88. The plot on right is the absolute count of colonies detected in each experiment. *n*_*Ctr*_ = 48 and *n*_*Li*_ = 40 show average colony count for control and light pictures respectively. The different colors used here are to show different pairs (1:control/2:light) of experiments.

It is also possible to see the change in area and count of CFUs after test experiments. In Fig. 4, the graph on the left shows the change in CFU areas after light irradiation. The control areas are normalized to 1. Each color is used to represent a different experiment. Overall, the CFU sizes are seen to decrease after light irradiation in this case. The graph on the right of Fig. 4 shows the absolute CFU count in both control and test conditions. It can be seen that the average count in this experiment session has decreased. In similar fashion, different images from control and test experiments can be analyzed in AutoCellSeg.

AutoCellSeg draws a priori information automatically from the input examples even if the colonies have a considerably high variation in size. Therefore, AutoCellSeg can easily be used for other data sets. This can work for different cell structures too, even when they are not very round, as AutoCellSeg takes eccentricity of objects into account using graphical a priori knowledge input. AutoCellSeg also extracts other a priori features based on intensity, in case of the detection of false positives. A video example of usage of AutoCellSeg for control/test pair analysis is given in Supplementary Data 4.

### Application to other data set

AutoCellSeg was applied to other image data sets with colonies. It is reasonable to try a data set of well segmented images with a different background and foreground in order to show the robustness of AutoCellSeg. Moreover, it is also imperative to demonstrate the performance of AutoCellSeg in different operation modes. The performance of AutoCellSeg on fluorescent mammalian cell segmentation and batch data and on randomly selected images was also shown (see Supplementary Data 5).

#### Inverted background

One challenge would be to use a data set having a light background with darker colonies in the foreground to see if the software can adapt well to the colonies. We used a data set provided at OpenCFU project website: https://sourceforge.net/projects/opencfu/files/samples/plosPicHQ.zip/download, which is based on *Staph*. *au*. on LB agar plates. There are 19 high quality images in this data set each with a resolution of 1538 × 1536. AutoCellSeg was compared with OpenCFU by running both of them in the partially automated mode (See ‘Process Selection’ in Supplementary Data 2).

The a priori knowledge for OpenCFU is based on trail and error and no overview of complete data set is available. OpenCFU was run with the minimum radius of 1 pixel (global parameter) for the colonies since several images have very small colonies. Parameter settings for AutoCellSeg are given in Supplementary Data 5.

**Figure 5:**
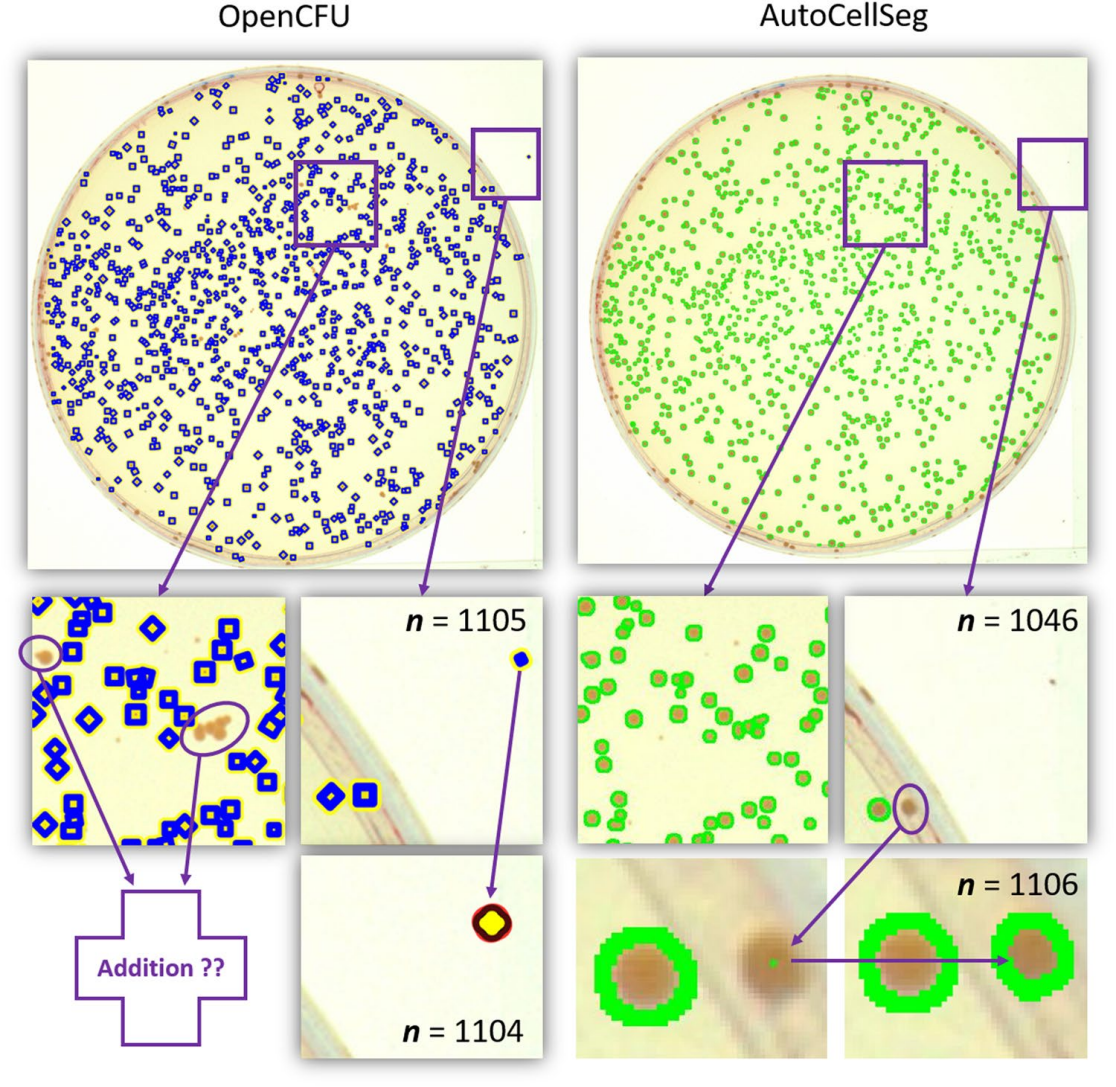
AutoCellSeg and OpenCFU segmentation results for the images with lighter background and darker colonies. The blue boxes (top-left image) show detection result from OpenCFU and green delineation (top-right image) is for AutoCellSeg segmentation. Zoomed-in sections of the selected image areas of images in first row without post-editing (second row). Result after correction step (last row). OpenCFU has missed a conglomeration of colonies (first image, second row). OpenCFU has detected some noise outside of the dish (second image, second row). AutoCellSeg segmentation of the same section as in first image of the second row (third image, second row). In AutoCellSeg, some colonies on the border of dish remain undetected (last image, second row). Erroneous segment detected outside could be deleted in OpenCFU (second image, last row). Undetected segments could be clicked upon interactively in AutoCellSeg (third image, last row). Based on the seed point defined by user, the undetected colony is segmented using fast marching method (last image, last row).

The results for one of the densely populated dishes are shown in Fig. 5. OpenCFU does a good job in finding almost all of the colonies in automated mode. The underestimation of colonies by AutoCellSeg in this case was caused due to the exclusion of the colonies on the boundaries of petri dish in the fully automated mode without correction. However, the ground truth was not at hand available from the link where this data set was downloaded. Therefore, it was only reasonable to compare the results of both software with each other. If we choose a very small value for lower radius in OpenCFU, noise outside the container is detected as shown in the second image of second row in Fig. 5. Here, AutoCellSeg has an advantage because the container mask is extracted automatically and nothing is detected outside of it. On the other hand, AutoCellSeg may leave some smaller colonies undetected depending upon the user selection during a priori extraction phase. But this is rarely the case and can easily be solved in the correction step.

In the post-processing, only the removal of false segments are possible in OpenCFU as indicated by arrow in second image of last row in Fig. 5. Therein lies a glaring problem, that in the case of undetected segments, a user cannot add new segments (see first image in second row of Fig. 5). In this regard, AutoCellSeg offers full flexibility by adding undetected and removing falsely detected segments (see third image in second and third row of Fig. 5). The aim here is to compare the colony count for all images by both software by taking into account the size of colonies detected. The final count obtained from both the software were quite comparable (see Supplementary Data 5).

## Discussion

AutoCellSeg provides new features and more flexibility in comparison to existing software tools for a better user experience (see end of Supplementary Data 5 for a comparison overview). This has been demonstrated on a new benchmark data set based on bacteria colonies and established benchmarks for cell segmentation in contrast to CellProfiler and OpenCFU, that also perform similar task of segmentation and counting. The methods used in AutoCellSeg are based on fuzzy a priori feature extraction for feedback based automatic tuning of parameters in watershed segmentation and post-processing.

AutoCellSeg comes with a GUI which allows the user to select images or an entire folder, change the parameters, choose the mode of operation, select the a priori information, correct the results graphically, create plots and save all the results. The entire pipeline of AutoCellSeg is shown in Supplementary Data 2. Depending upon the amount of data, the degree of precision required and the level of difficulty in segmentation, a user is free to select a fully automatic approach, where post-editing occurs after all the images have been segmented, a semi-automatic mode, where post-editing is done after each individual image or manual labeling, which involves the drawing of each boundary by using freehand drawing, circles or seed points.

The post-editing step involves removing unwanted segments and adding new segments by using seed points graphically inserted by the user. To maximize the quality of results, it is advisable to follow the steps given in Supplementary Data 2 for each operation mode. The software delivers masks, outlined images,.csv file with counts and sizes, size density distribution, summary plots and the chosen parameters after each run. The results can then be saved to the local drive by clicking the *Save results* button. The parameters and options that the user can change are explained in detail in Supplementary Data 3.

The main advantage in using AutoCellSeg lies in its ability to adjust the results in an intuitive and interactive fashion using simple graphical selection. It is versatile and robust enough to cope with different image variations and to adapt to various backgrounds and cell morphologies in a variety of data sets. Another attractive feature in AutoCellSeg enables to compare the results statistically between control/test image pairs using size density distribution of cells/colonies on top of plots based on the cell/colony count and normalized size. Additional segment features like eccentricity, radius and mean intensity are also calculated and optionally could be saved locally using.csv file. It can also be used as a labeling tool manually using freehand delineation, shapes like ellipses or automatically using fast marching method.

AutoCellSeg performs well on different image data sets. It is not only useful for segmentation of bright field images but also for cell images using fluorescent light. This has been elaborated using benchmark data set of mammalian cells i.e. BBBC008 containing human HT29 colon-cancer cells (see ‘Cell Segmentation’ in Supplementary Data 5). Based on several evaluation measures, AutoCellSeg was compared with OpenCFU and was shown to perform better than OpenCFU.

Since AutoCellSeg may run slower than OpenCFU in processing, it may be useful to implement the image processing algorithms on graphical processing units in the future. Moreover, selection of a variety of object features from the input panel such as mean intensity, solidity, etc. in addition to user-defined features will also be included. This would give user more flexibility to choose the a priori knowledge for segmentation. New detection and segmentation methods, such as semantic segmentation using deep learning, will also be implemented in future. Code writing of AutoCellSeg in other languages such as Python etc. is also very useful in order to allow the user to extend the program in different programming languages.

## Methods

AutoCellSeg is based on fuzzy a priori information (see the next Sub-section for more details) extracted from the user’s selection using the fast marching method^25^ (see Supplementary Data 2 for more details). This information relies on the size *a* and optionally the intensity *b* of the selected cells/CFUs and serves as the base for the supervised multi-threshold image segmentation. The input image **I**_*in*_ is first pre-processed based on channel information. It is then passed on to an adaptive threshold segmentation step, where a binary mask is created containing the CFUs/cells in form of Binary Large Objects (BLOBs) **b**_*i*_. The segmentation results, one for each intensity threshold, are overlapped iteratively over a search space of successive threshold values between 0 and 1 (see Section 6). A BLOB plausibility check is performed over the resulting image where any **b**_*i*_ having size *a*_*i*_ < *a*_*min*_ (*a*_*min*_ is derived from fuzzy a priori knowledge as described in Section 5) is removed. Furthermore, bigger BLOBs with *a*_*i*_ > *a*_*big*_ (where *a*_*big*_ is the upper 30% of median *a*_*i*_) are passed onto feedback-based watershed segmentation step which adapts the value of H-maxima transform for regional maxima of **I**_*in*_^26^. The regional maxima are used as seed points for watershed segmentation in the automatic mode of AutoCellSeg (see Section 6). After this step, feature extraction of each segment is done and the segment features can be saved in an external.csv file. The user is then able to correct the results if necessary, which is also done with the fast marching method (see Supplementary Data 2).

### Fuzzy a priori information

**Figure 6:**
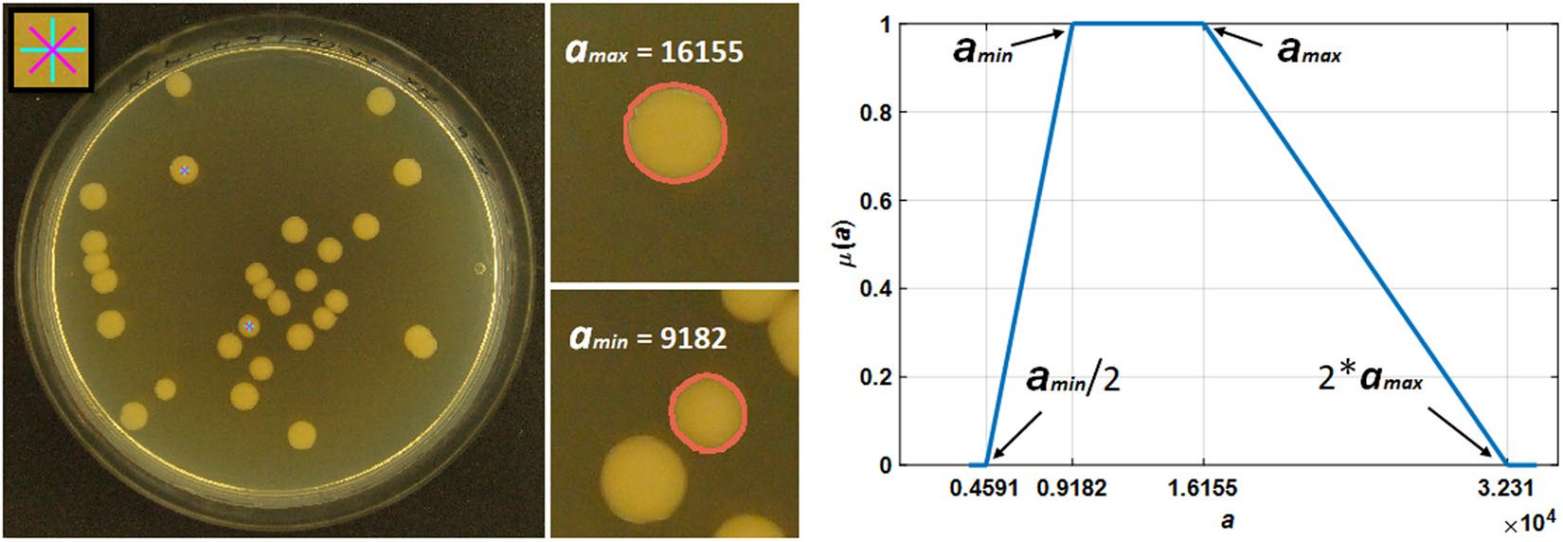
The extraction process of a priori information from the AutoCellSeg GUI. The asterisk sign on the top left corner of the full RGB image shows the selection marker. The initial colonies could be selected by left-clicking of the mouse. The two colonies selected in the full RGB are indicated by the asterisk signs. The two smaller images in the middle show the detection of these colonies using the fast marching method (indicated by the pink colored delineation). The maximum and minimum colony sizes are then used for the extraction of a priori knowledge as shown in the graph on the right where *μ* (*a*) (fuzzy membership function) is on y-axis and *a* (colony sizes) is on x-axis. The edges of the trapezoid are indicated by black arrows. The edges have the values (0.5*a*_*min*_, *a*_*min*_, *a*_*max*_, 2*a*_*max*_) = (4591, 9182, 16155, 32310).

As the method is supervised, a priori information is required. Currently popular GUIs like CellProfiler and OpenCFU require the user to input the a priori information manually or by moving a sliding bar. However, this is not an intuitive selection as the user has no foreknowledge about the needed parameters. Moreover, the precise size and circularity factor is also not easily extracted just by looking at the image. Therefore, a less experienced user selects the initial a priori by a trial and error method. In AutoCellSeg, the information is extracted automatically by a simple mouse click on the CFU in the image displayed in the GUI. The a priori area *a*_*a*|*i*_ is calculated automatically using the fast marching method from these selected BLOBs *b*_*a*|*i*_. The mean intensity information *b*_*a*|*i*_ is also calculated from *b*_*a*|*i*_. This process is shown in Fig. 6. The center of the colorful mark is the user input as shown in the top left corner of Fig. 6. Using this center, a BLOB is detected. It is recommended to select a small and a large colony/cell from the input images. In this example (Fig. 6), a small and a large CFU were selected to extract a priori information. The detection of BLOBs from the graphical input by the user is shown in the subfigures on the right hand side of Fig. 6. The pink outlines are the boundaries of the BLOBs. The area features (i.e. *a*_*min*_ = min (*a*_*a*|*i*_) and *a*_*max*_ = max (*a*_*a*|*i*_)) are calculated for both of them. Thereafter, a fuzzy trapezoidal membership function *μ* (*a*) (according to^26^) is used. The edges of trapezoid are (*p*_1_, *p*_2_, *p*_3_, *p*_4_) = (0.5*a*_*min*_, *a*_*min*_, *a*_*max*_, 2*a*_*max*_. This is shown in the plot of Fig. 6. If the a priori intensity selection is activated from options dialog, then only the BLOBs having an intensity values between [*b*_*min*_, *b*_*max*_] are kept, where *b*_*min*_ = 0.5 min (*b*_*a*|*i*_) and *b*_*max*_ = min (1,1.5 max (*b*_*a*|*i*_)).

### Multi-threshold segmentation

The automatic multi-threshold segmentation runs over the search space of intensity threshold *t* of an image **I** with pixel values *I*_*mn*_ where *m* and *n* are the row and column indices respectively. In our case, *t*_*min*_ < *t* < *t*_*max*_ where *t*_*min*_ = 0.01 and *t*_*max*_ = 0.99 as we normalize our image **I** between 0 and 1 according to: ${I}_{mn,norm}=\frac{{I}_{mn}-{I}_{mn,min}}{{I}_{mn,max}-{I}_{mn,min}}$ where, *I*_*mn*,*min*_ and *I*_*mn*,*max*_ are the minimum and maximum pixel values of **I** respectively. An incremental step *δ* can be varied to alter the outcome. Choosing low *δ* values can fine-tune the results but would cost more time and vice versa. We chose *δ* = 0.1 but more experienced users can also change it manually in the option dialog (see Supplementary Data 3) according to their application and type of data. At each *t*_*i*_, with $i\in (0,\frac{{t}_{max}-{t}_{min}}{\delta })$ using *t*_*i*_ = min (*δ* · *i* + *t*_*min*_, *t*_*max*_), a segmentation **I**_*t*,*i*_ is obtained. After all the threshold levels are run, the results are gathered in **I**_*t*,*all*_ using logical OR operator.

### Feedback-based automatic watershed segmentation

**Figure 7:**
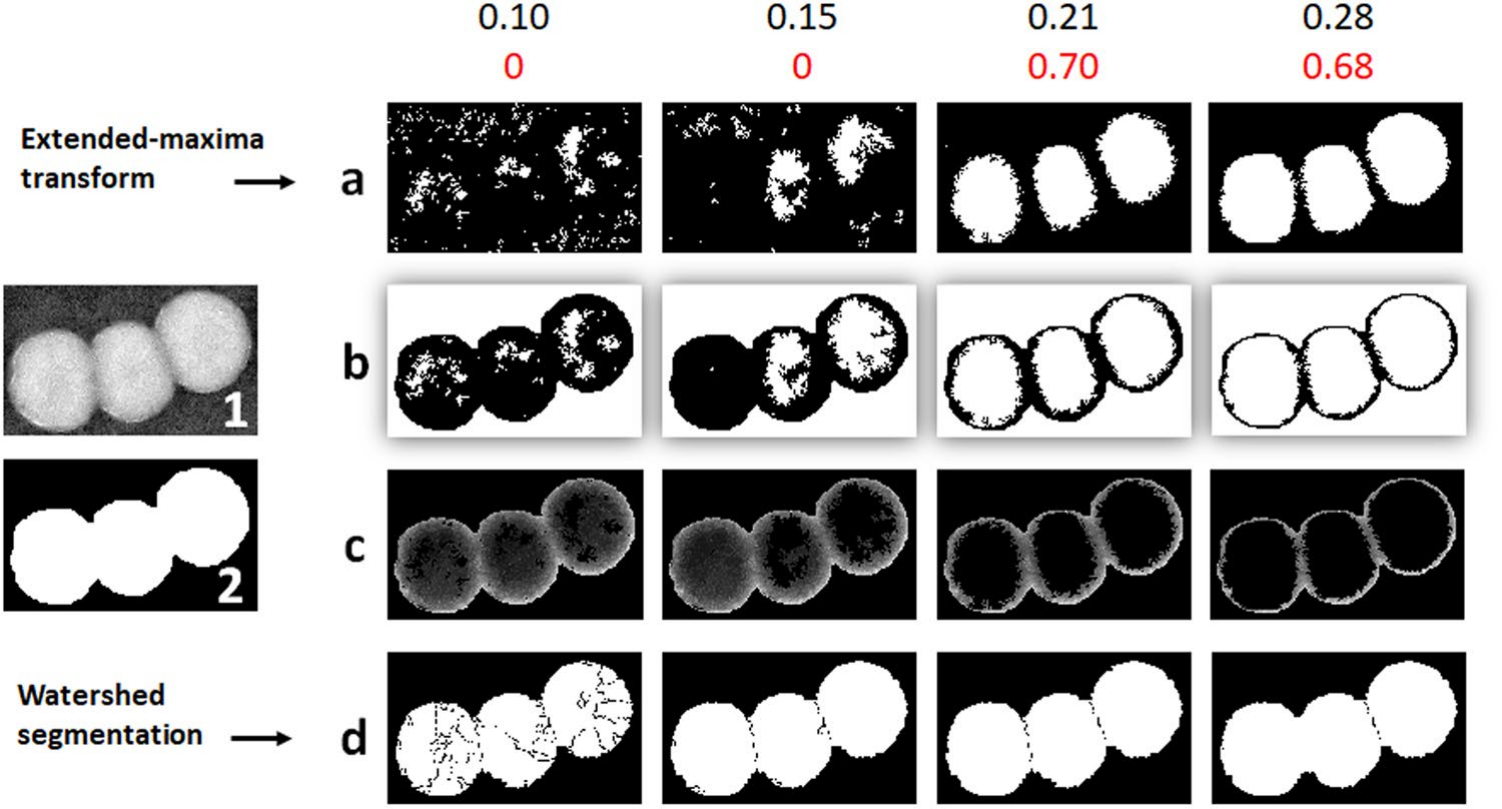
The parameter adaptation for *h* using criteria (1) and (2). Image **1** is the original grayscale image of the chunk of colonies and image **2** represents the initial BLOB detected. Values in black are the H-maxima transform (*h*_*i*_) and red values represent the quality of segmentation (*Q*_1_ (*h*_*i*_)). Row (**a**) shows the result of applying hole filling operation after using extended-maxima transform at different *h*_*i*_. Row (**b**) shows just the representation of selected seed points using an OR operation of seed points image with the complement of the mask. Row (**c**) shows the result of imposing minima operation on complemented grayscale image and the OR combination of complemented mask with the seed points image. The final result of applying watershed algorithm from the image obtained from preceding step is shown in the last row (**d**). Here, *h*_*i*_ = (0.1, 0.15, 0.21, 0.28) were selected to show the affect of using different *h*_*i*_ on the quality outcome *Q*_1_ (*h*_*i*_). It can be seen from this example, that the best result *Q*_1,*opt*_ = 0.70 is obtained at *h*_*i*_ = *h*_*opt*_ = 0.21.

This method has been adapted from^26^, where it was applied to adapt intensity threshold for segmentation. The watershed segmentation^27^ is quite useful in separating chunks of BLOBs. It separates a bigger chunk based on the defined *catchment basins* or the seed points using the intensity information where dark pixels represent low elevation and vice versa. However, detecting the seed points may not be straight-forward as objects to be segmented have varying intensity maxima. Choosing different values of H-maxima transform *h* of intensity produces different results. Therefore, we tune the parameter *h* iteratively by feedback-based information using a quality criterion *Q*_1_, such that *h*_*min*_ ≤ *h*_*i*_ ≤ *h*_*max*_. We kept ${h}_{min}=0.10$ and *h*_*max*_ = 0.3 using a step size *δ* = 0.01. However, the parameter range for *h* is flexible and user can alter it according to his requirements. The parameter *h* is adapted according to:

1 $${h}_{opt}={\rm{\arg }}\,\mathop{{\rm{\max }}}\limits_{{h}_{i}}{Q}_{1}({h}_{i})$$

2 $${Q}_{1}={\mu }_{1}\cdot {\mu }_{2}$$

where, *μ*_1_ and *μ*_2_ are fuzzy trapezoidal membership functions for evaluation of expected count and size respectively. For *μ*_1_, the trapezoidal edges are (*p*_1_, *p*_2_, *p*_3_, *p*_4_) = (1, *n*_*x*_, 2*n*_*x*_, 3*n*_*x*_ − 1). The variable *n*_*x*_ is extracted using the size *a*_*i*_ of BLOB **b**_*i*_ having pixel values equal to *b* (*m*, *n*) (*m* and *n* are row and column indices respectively) such that: ${n}_{x}=\frac{{\sum }_{m=1}^{M}{\sum }_{n=1}^{N}b(m,n)}{{a}_{min}},$ and *μ*_2_ is trapezoidal membership function for size distribution with edges (*p*_1_, *p*_2_, *p*_3_, *p*_4_) = (0.5*a*_*min*_, *a*_*min*_, 2*a*_*min*_, max (2*a*_*min*_, *a*_*max*_)). where, *Q*_1_ (*h*_*i*_) is the quality of segmentation outcome at each *h*_*i*_. An example is given in Fig. 7, that explains the feedback-based parameter adaptation for the watershed segmentation in detail.

### Software/data availability

Tool with source code and the data used in obtaining results presented in this paper are freely available at: https://github.com/AngeloTorelli/AutoCellSeg. It was implemented using MATLAB2016b under Windows 10 Pro and tested for different operating systems.

## Electronic supplementary material

Supplementary Material
