# Supplementary material for: AutoCellSeg: robust automatic colony forming unit (CFU)/cell analysis using adaptive image segmentation and easy-to-use post-editing techniques
Source: Sci Rep. 2018 May 8;8:7302. doi: 10.1038/s41598-018-24916-9 (PMC5940850; doi:10.1038/s41598-018-24916-9)
Supplement: Supplementary file 1 — Supplementary Material [file 41598_2018_24916_MOESM1_ESM.pdf]

AutoCellSeg: robust automatic colony forming unit (CFU)/cell analysis using adaptive image segmentation and easy-to-use post-editing techniques

Arif ul Maula Khan[1,*], **Angelo Torelli**[1,2], **Ivo Wolf**[2], **and Norbert Gretz**[1]

[1]Medical Faculty Mannheim, Medical Research Center, University of Heidelberg, Mannheim, 68167, Germany
[2]Mannheim University of Applied Sciences, 68163 Mannheim, Germany
[*]arifulmaula.khan@medma.uni-heidelberg.de

Supplementary data 1

The quality criterion Q used in this work is based on:

1. Object count (q_1): It is defined as:

$$q_1 = \frac{|\text{card}(\mathscr{T}) - \text{card}(\mathscr{S}_c)|}{\text{card}(\mathscr{T})} \tag{1}$$

with,

$$\mathscr{S}_c = \{\mathscr{S}_u | \max(\text{card}(\mathscr{S}_u \cap \mathscr{T}_1), \ldots, \text{card}(\mathscr{S}_u \cap \mathscr{T}_z)) > \frac{1}{3} C_u\}. \tag{2}$$

Here, S is the set of all segmented BLOBs (Binary Large Objects) such that, $\mathscr{S} = \{\mathscr{S}_u | u = 1, ..., U\}$ and U is the total number of segmented BLOBs. $\mathscr{S}_u$ is a set of C_u pixel values and is defined as: $\mathscr{S}_u = \{\{S_{ux,1}, S_{uy,1}\}, \cdots, \{S_{ux,C_u}, S_{uy,C_u}\}\}$. Here, x and y are representing image coordinates and u represents a given BLOB index. Altogether, $S_{ux,1}$ and $S_{uy,1}$ represent the row and column pixel indices of $\mathbf{S}_u$. $\mathscr{T}$ is the set of all the ground truth BLOBs such that, $\mathscr{T} = \{\mathscr{T}_v | v = 1, ..., V\}$ and V is the total number of ground truth BLOBs. Here, $\mathscr{T}_v$ is defined as $\mathscr{T}_v = \{\{T_{vx,1}, T_{vy,1}\}, \cdots, \{T_{vx,C_v}, T_{vy,C_v}\}\}$ where, C_v is the number of pixels in T_v.

Th overlap of $\mathscr{S}_u$ and $\mathscr{T}_v$ is calculated. If it exceeds $1/3$ of $\mathscr{T}_v$, BLOB is added to the set containing correct segmented objects $\mathscr{S}_c$ and deleted from the ground truth set such that for a new $\mathscr{S}_u$, z = 1, ... , V - n_v (number of $\mathscr{T}_v$ BLOBs affected by overlap) and z is new number of remaining T_v elements.

2. Pixel misclassification (q_2): This measure is like Rand Index and is described as:

$$q_2 = \frac{\sum_{i,j} |\text{sign}(\delta_{\text{truth},ij} - \delta_{\text{seg},ij})|}{\sum_{i,j} \text{sign}(\delta_{\text{truth},ij} + \delta_{\text{seg},ij})}. \tag{3}$$

The fuzzy function μ that we used in this case is monotonic with adjustable higher and lower bounds α and β and is given as:

$$\mu(\theta; c, \alpha, \beta) = \begin{cases} 1, & \theta \leqslant \alpha \\ 1 - 2^{c-1}(\frac{\theta-\alpha}{\beta-\alpha})^c, & \alpha < \theta \leqslant \frac{\alpha+\beta}{2} \\ 2^{c-1}(\frac{\theta-\beta}{\beta-\alpha})^c, & \frac{\alpha+\beta}{2} < \theta < \beta \\ 0, & \theta \geqslant \beta \end{cases} \tag{4}$$

The parameter c is used to define the curvature of the function, we used $c = 2$ i.e. a spline based function for evaluation. α and β were chosen to be 0 and 1 respectively such that:

$$\mu_1 = 1 - \mu(q_1; 2, 0, 1). \tag{5}$$

$$\mu_2 = 1 - \mu(q_2; 2, 0, 1). \tag{6}$$

Using criteria (5) and (6) , the overall segmentation quality measure is given as:

$$Q = \mu_1 \cdot \mu_2. \tag{7}$$

The combination presented in criterion (7) is valid for binary ground truth objects.

Supplementary data 2

Prerequisites

The only requirements the user has to fulfill are:

- The image file types should be in the following formats: JPG, PNG, TIF or BMP.
- To create comparison plots the selected image should contain the test or control name defined under *Change parameters* (see Fig. 5) in their file names. If the name consists of different names and details regarding the experiment, please separate these with a space (' '). An underscore ('_') and a period ('.') and other symbols have yet to be implemented.

After starting AutoCellSeg, the user is able (but not required) to change the parameters and other options by opening the *Change parameters* dialog (see Fig. 1). This is activated by clicking on the *Change parameters* button (marked in Fig. 1 with 'G'). If the user wishes to change any parameters or options, this should be done before a process has started otherwise the changes will not be taken into account.

Data selection

The user then has to add the images that need to be processed by AutoCellSeg. Here, the user has two options:

- Add any number of images by clicking on the *Select images* button (marked in Fig. 1 with 'A'). Here, an open file dialog box will appear where the user has the following options:
  - Click to select an individual image.
  - Multiple selection is done by holding down the Shift or Ctrl key and clicking on the wanted images.
- Add all the images inside a folder by selecting the directory itself.

The images the user has selected are then displayed on the main display panel of the software (marked with a red frame in Fig. 1). The number of images can range from 1 to any number (*Create plots* button can only display results of maximum 60 images) but only up to 20 images are displayed at once in the display panel. To view the other images, the user has to click on the navigation buttons previous page ('<') or next page ('>') (marked in Fig. 1 with 'I' and 'J' respectively). By clicking on an image, the image will be displayed in a separate window, which can be maximized to the screen size of the user. A user can zoom in and out of each individual image to see more details.

Process selection

The entire pipeline of AutoCellSeg is given in Fig. 2. The user can choose between three processes. Each one has a varying level of interaction with the user whereby *Fully automated* has the least, and *Manual selection* has the most interaction steps. Press the *Run* button (marked in Fig. 1 with 'C') to activate the selected process, i.e. *Fully automated*, *Partially automated* or *Manual selection* (marked in Fig. 1 with '*a*', '*b*' and '*c*' respectively).

Fully automated: is the only process where the requirement needs to be satisfied before the process runs. The requirement consists of selecting the a priori knowledge from all selected images. The following steps should be considered in this regard:

- Select a small and a big colony in any of the images loaded to AutoCellSeg. It is also possible that one of the selected images has both the biggest and the smallest colonies. In such a case, the user does not have to open other images. To maximize the quality of the results, the user should follow some case scenario approaches and various methods of selection described later.

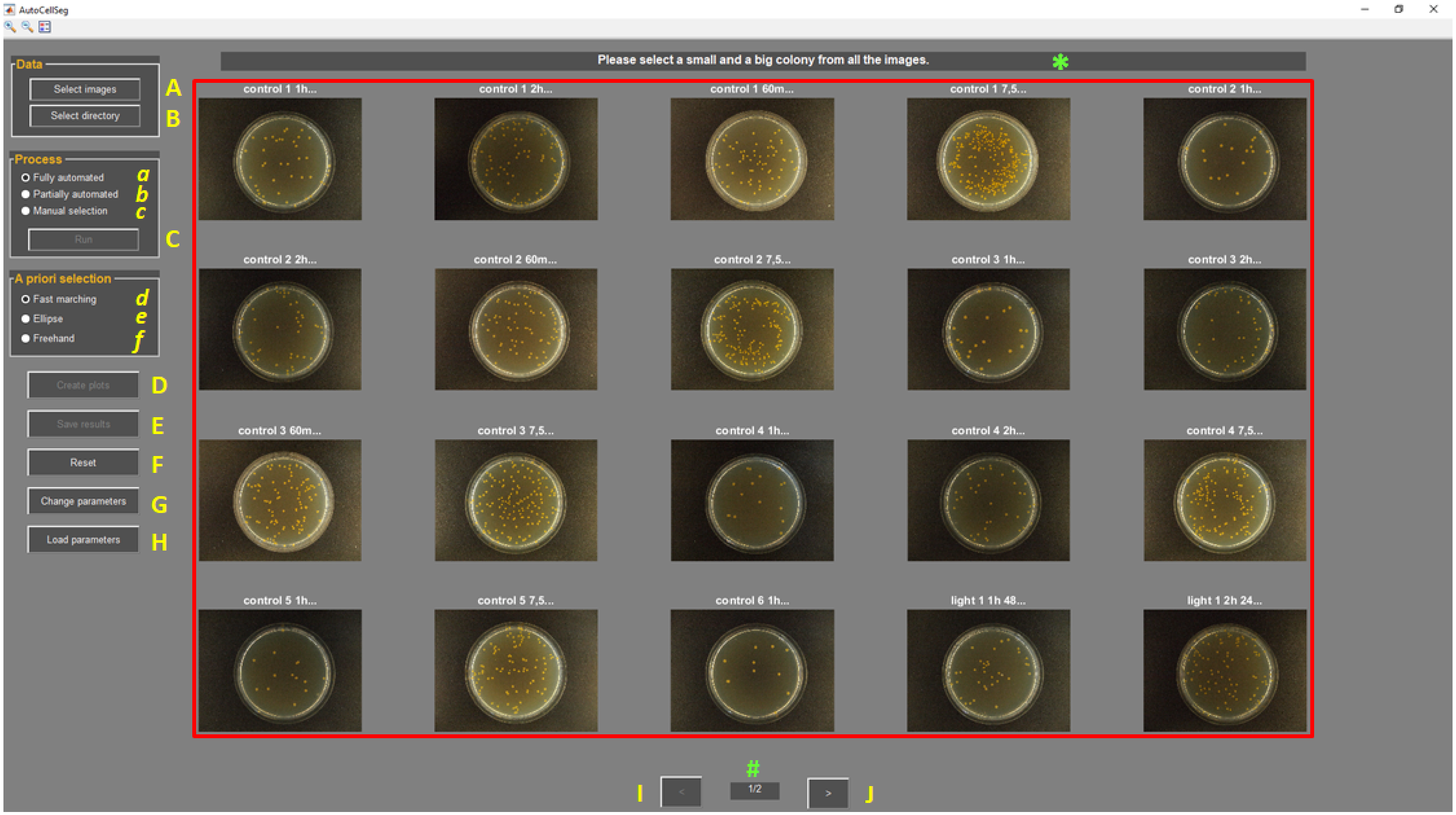

Figure 1. Layout of AutoCellSeg. Upper case letters represent buttons, lower case letters represent radio buttons options, thr red frame shows the main display panel, and the green symbols represent labels/status.

- When the user is satisfied with his selection, he can activate the fully automated process by pressing the *Run* button. This processes all of the images without any additional interaction with the user. It is computationally expensive and, therefore, can take several minutes to finish, depending upon the number of images and criteria selected.

A status bar shows the running progress of the process as shown by the asterisk in Fig. 1.

Partially automated: is similar to fully automated but instead of selecting the a priori for all the images, it requires a priori information from each individual image. The following are the steps to be followed in the *Partially automated* process:

- Press the *Run* button to start the process. AutoCellSeg then opens one image after another until all the images are processed. Every time an image is opened, the user is asked to:
  - Select a small and a big colony in the image. To maximize the quality of the results, the user should follow some case scenario approaches and various methods of selection.
  - After the image has been processed, the user has one chance to correct the results before it continues to the next image. This will not be the last time the user is able to correct the image, since he will be given another opportunity to do so after all the images are processed.

Manual selection: involves the manual labeling of all the images where the user has to select every single colony in each image manually. This is the process with the highest user interaction. The steps for the manual selection are:

- Press the *Run* button to start the process. AutoCellSeg then opens one image after another until all the images are labeled. Every time an image is opened, the user is asked to:

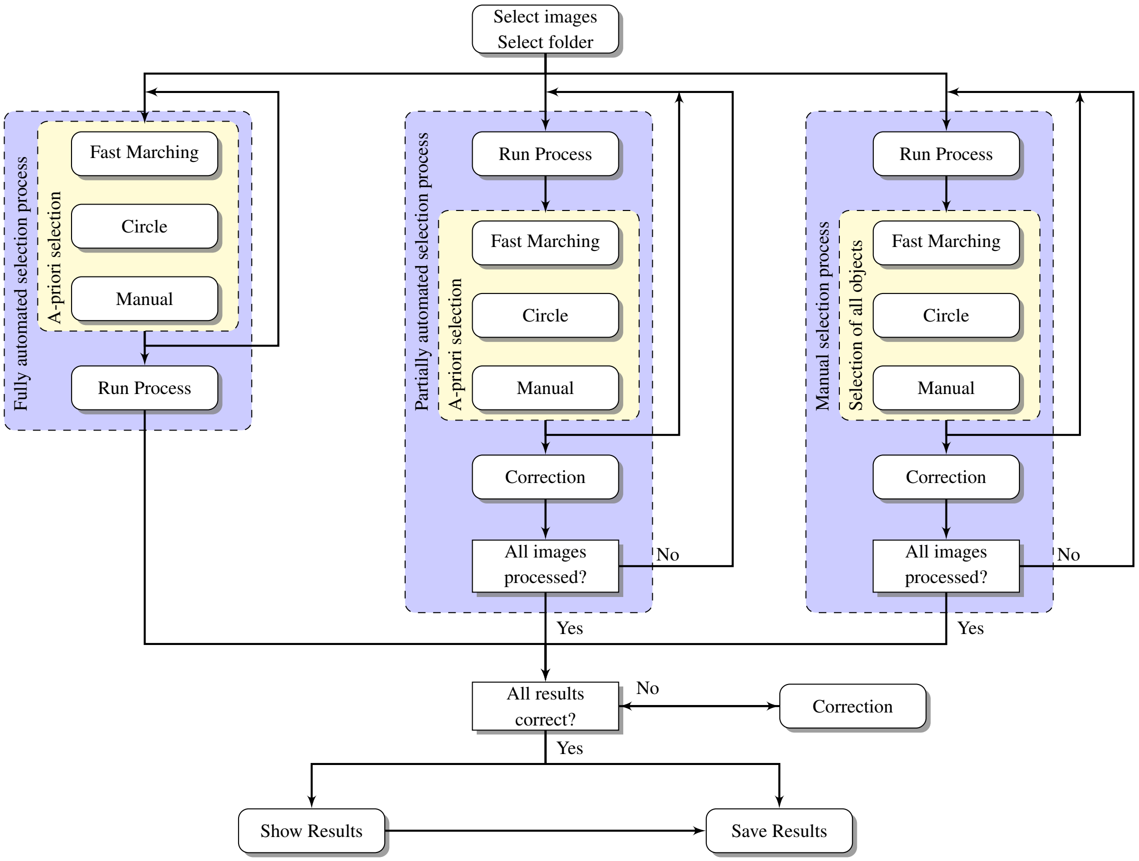

Figure 2. The pipeline for AutoCellSeg showing each individual process: 1) Fully automated, 2) Partially automated, and 3) Manual labeling. Each process is shown using a blue colored box. The yellow boxes inside the blue boxes indicate different a priori selection methods that the user can choose from. After the process, the user can correct the results by adding new or removing unwanted segments. When the user is satisfied with the results, the user can then create plots for comparisons between experiments and also save the results.

- Select every colony on the image. Like in the other two aforementioned processes, the user has various methods of selection described in following sub-section.
- After all the colonies have been selected, the user has a one time chance to correct the results before it continues to the next image. Eventually, when all the images have been processed, the user can correct each individual result again.

After all the images are processed independently of the process method, the user has the chance to correct the results, create plots and save all the results.

A priori selection method

AutoCellSeg allows the user to choose between one of the following selection methods:

Fast marching method: uses the built-in MATLAB function[1] that implements the fast marching method[1]. Here, the logical seed points are selected interactively by clicking on the region inside the object boundaries to create a mask. This mask is used in conjunction with the weight array based on gray scale intensity differences[2]. The parameter used to affect the outcome here is a threshold value that can range between 0 and 1. This value specifies the level at which the function thresholds the normalized geodesic distance map of the fast marching method to obtain the output binary image. AutoCellSeg allows the user to select the points in the image with the mouse pointer as follows:

- Use left clicks to select seed points.
- Press 'Backspace' or 'Delete' to remove the previously selected seed point.
- To finish selecting seed points by adding a final seed point, press shift-click, right-click, or double-click.
- To finish selecting seed points without adding a final seed point, press 'Return/Enter'.

Ellipse selection: uses an internal MATLAB function which gives the user the possibility to draw an ellipse interactively. Click and drag to specify the size and position of the ellipse. The ellipse also supports a context menu that one can use to control aspects of appearance and behavior.

Freehand drawing: uses an internal MATLAB function which allows the user to draw a freehand region. Click to start and drag to draw the freehand region. This function draws a straight line connecting the last point the user draws with the first point.

The user does not have to select the smallest or the biggest colony but it is recommended to select one of the smallest and one of the biggest. For this, the overview of all images can help the user decide which of the images contains approximately the smallest or biggest colonies. The user is also not restricted to selecting only two colonies. In this case a larger selection is advantageous, especially when the user is not sure about the extreme sizes between all the colonies. In some scenarios, it is recommended to follow the approaches given in (Supplementary video 1):

- Colonies with anomalies in shape or size should not be selected for the a priori, but could later be added in the correction stage, if not already recognized by the algorithm.
- A similar recommendation can be made with conglomerates which should be selected in the correction phase, rather than in the a priori phase.
- Another problematic CFU type is the one lying very close to the petri dish borders. It should also be added in the subsequent correction stage or added with the ellipse or manual selection instead of the fast marching selection.
- When a selection is inaccurate or the colony is not fully segmented, the wrong a priori information could skew the results. When this happens, the user can either select additional colonies or rerun the selection step and select the same colony on a different spot or on multiple spots.

Correction

After the process has finished running, the user has the chance to correct the results of each image by clicking on the image thumbnail displayed on the main GUI. The image is then opened in a new window where the user is able to execute the following manipulations:

- Left click to add a new segment.

[1] The built-in MATLAB function *imsegfmm* was used.
[2] The built-in MATLAB function *graydiffweight* was used.

- Right click to delete an existing segment.
- Middle click to update the overlaid display image and the colony count in the title.
- Closing the window to end the process.

Akin to a priori selection, it is recommended to follow a certain approach when encountering a specific scenario (see Supplementary video 1):

- Additional recognition: is when some background is recognized in addition to the colony, then the colony needs to be deleted and added again.
- Partial recognition: is when a colony is not recognized fully. The user can either set a seed point to the border of the recognized segmentation to enlarge the boundary or delete the colony, add it again, and if needed do the aforementioned mentioned.
- Extremes: are cumbersome colonies that are difficult to recognize correctly. It is very likely that some of the petri dish (or similar background) can be recognized as part of the colony. The user can try to add it but the results could skew the statistics shown in the plots. It is the user's discretion to keep or to discard it.
- False negative:
  - in a single colony: can be solved by adding the colony and adjust it by setting a seed point to the border of the recognized segmentation
  - in a conglomeration: can be solved by deleting all recognized colonies of one conglomeration and adding seed points at the center of each colony (expert user is required) that forms that conglomeration.
- False positive: are falsely recognized colonies that can be deleted.
- Falsely identified conglomeration: occurs when the intensity of one colony varies a lot, or when its size is very big. Here, the user can either delete all the segments and add the individual colony again, or add seed points between the borders of the falsely recognized segments to merge them into one.
- No separation or wrong separation of conglomerates: must be solved in the same way as false negative in a conglomeration. By deleting all recognized colonies of the one conglomeration and adding as many seed points (at the center of each colony) as the number of colonies forming that conglomeration.

Internally the adding function in the correction phase of AutoCellSeg is processed in almost the same manner as the fast marching method used for a priori selection. The only difference is that instead of giving all the seed points at once and running the function through the entire image, this function uses only one seed point at a time and runs it on a cropped part of the image which is of the same size as the biggest selected colony in the a priori selection phase. Moreover, the threshold value is set to 0.02 as a default. The threshold parameter for the addition of new segments with fast marching can be changed in the options dialog. To demonstrate the effect of the different values for the threshold parameter a two colony aggregate example was used as shown in Fig. 3.

Create plots

The user can click on the *Create plots* button, only if the requirements are met and after the software has processed all the images. This button will create the following results:

- An overlaid KDE plot for all experiments using a specified bandwidth bw.
- Absolute sizes of colonies for all experiments.
- A plot showing the change in absolute colony sizes normalized to 1.
- A CFU count plot for a comparison between both test and control images in each individual case.

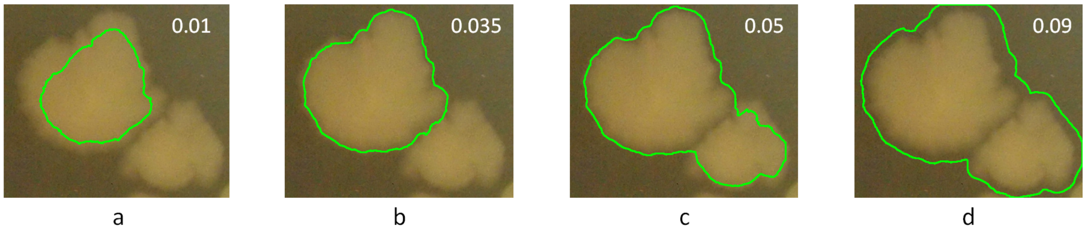

Figure 3. The outcomes of different threshold values in fast marching method. In the given image, two adjacent colonies are present. If a user wants to segment only one of them (e.g. the bigger one) by a single-click segment addition feature of AutoCellSeg, the threshold for fast marching method has to be set properly. With a value of 0.01, the selected colony is not segmented fully as shown in (a). For a value of 0.035, the colony is segmented almost perfectly but some of the border on the left side is not included as shown in (b). Increasing the value to 0.05 creates a perfect contour of the colony but part of the other colony is wrongly added to the segmentation as seen in (c). At the threshold of 0.09, both colonies are detected as one aggregate as shown in (d).

Save results

By clicking on the *Save results* button (marked in Fig. 1 with 'E'), which is activated as soon as a process has finished running, AutoCellSeg will save all the results that include:

- An outlined overlaid image .
- A binary mask of the detected and fully filled segments (as white or ones) for each image.
- A csv file for the summary of features. This shows the image name, the number of colonies (colony count), the mean area, the mean radius and the mean eccentricity.
- A csv file with the experiment number and the ID, the size, the minor axis length, the eccentricity, the mean intensity and radius of the each colony.
- And all the plots created by the user using the *Create plots* button.
- Parameters used for the segmentation/analysis.

Additional information

The *Reset* button (marked in Fig. 1 with 'F') brings AutoCellSeg to a clean state and closes all the open figures. AutoCellSeg works on all the main operating systems and was fully tested only on Windows [3].

The *Load parameters*, indicated by 'H' in Fig. 1, enables the user to load the saved parameters in order to reproduce results previously obtained.

Other features to be included in future are:

- Selection of a variety of object features from the input panel such as mean intensity, solidity, etc. User-defined features will also be included.
- New detection and segmentation methods, such as deep learning, will be implemented in future.
- Implementation of AutoCellSeg in other languages, such as Python to allow the user to extend the program.
- Application to variety of data sets.

Image analysis pipeline

The image analysis pipeline involves pre-processing, segmentation, feature extraction and post-processing. The outcome of the individual steps are show in Fig. 4 and the steps are described sequentially as follows:

1. Input image $\mathbf{I}_{in}$ is read into AutoCellSeg.
2. Two or more colonies are selected using mouse left-click in AutoCellSeg GUI. The segment selection method is described in detail in Supplementary data 1.

[3] https://github.com/AngeloTorelli/AutoCellSeg

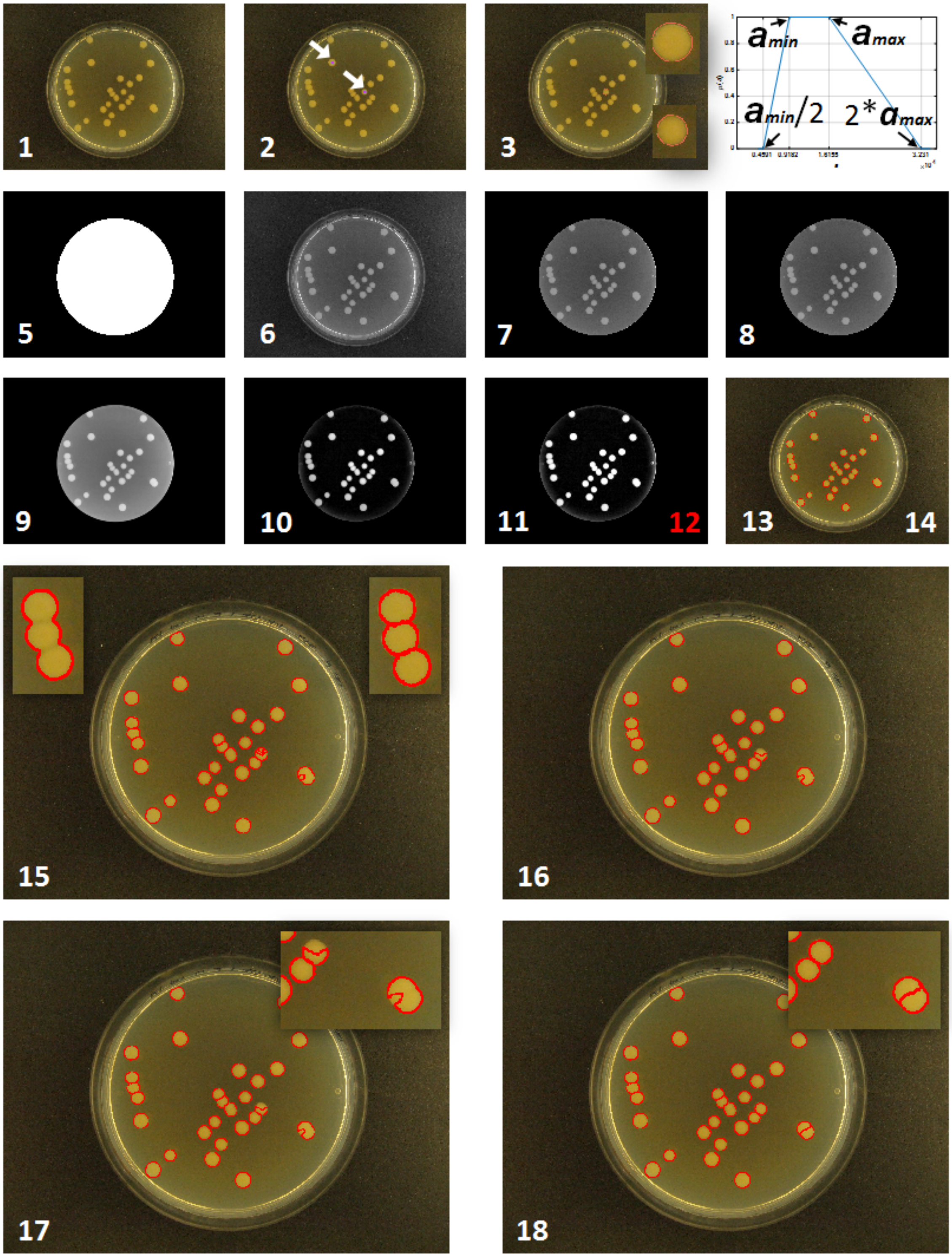

Figure 4. The outcome of each individual step of the image processing pipeline (step 1 to 18). Each image is indicated by a step number which corresponds to the aforementioned steps described in text with the same number. The red colored step indicates that it has not been turned on or used during this particular example.

3. The selected objects are segmented with the fast marching method to get an initial estimate about size and circularity features.

4. A priori information of size i.e. minimum size (a_{min}) and maximum size (a_{max}) is extracted along with the average eccentricity (e_m) of the selected segments

5. If $\mathbf{I}_{in}$ contains a CFU container, it is detected automatically using a binary mask $\mathbf{B}$ based on histogram information [4]

6. $\mathbf{I}_{in}$ is converted into a grayscale image (if it has more than one channel). The selection of the channel can be done either manually or automatically. It can also be either a specific channel or the mean of all channels.

7. The resulting grayscale image is then multiplied with the mask $\mathbf{B}$ to obtain $\mathbf{I}$.

8. $\mathbf{I}$ is then normalized between 0 and 1 using minimum and maximum intensity values to get $\mathbf{I}_{norm}$.

9. A Gaussian filter of size $s \times s$ (defined by user) is applied to $\mathbf{I}_{norm}$ and the resulting image $\mathbf{I}_{gauss}$ is then used for outliers removal.

10. 1 % quantiles are removed from lower and higher sides of intensity histogram to obtain $\mathbf{I}_o$.

11. $\mathbf{I}_c$ is obtained by performing background removal using morphological top hat filtering with disk of radius r as a structuring element.

12. At this stage, histogram equalization is done on $\mathbf{I}_c$, if specified by the user at the beginning, to get $\mathbf{I}_{eq}$. $\mathbf{I}_{eq}$ is equal to $\mathbf{I}_c$ if user sets the option of adapting histogram to false in the *Options* dialog (see Supplementary data 3).

13. $\mathbf{I}_{eq}$ is then segmented using multiple intensity threshold mechanisms to get $\mathbf{I}_{t,all}$ by gathering BLOBs at each t.

14. $\mathbf{I}_{t,all}$ is checked for plausible BLOBs to be passed on to automatic feedback-based watershed step. The plausibility criteria are:

    - if a BLOB $\mathbf{b}_i$ has $e_i > 0.5$, and
    - if a BLOB $\mathbf{b}_i$ has $a_i > a_{max}$.

15. BLOBs satisfying the criteria are processed through seed point guided watershed segmentation. Here, the seed points are controlled by regional maxima of the H-maxima intensity transform. However, this step is not required if seed points are defined externally as in the case of post-editing in AutoCellSeg.

16. The resulting image $\mathbf{I}_{ws}$ is then checked for plausibility criteria to exclude more eccentric and small segments based on a priori knowledge.

17. The graphically selected segments are kept if the corresponding segments detected are smaller than 10% of area encapsulated by initial segments to get $\mathbf{I}_{ws|i}$.

18. $\mathbf{I}_{ws|i}$ then enters the correction phase which includes the manual addition of undetected segments and removal of erroneous segments. This post-editing consists of the following steps:

    - In the segment removal step, the coordinates of graphical inputs are compared to the center coordinates of their nearest segments. The segment closest to an input is then deleted. For each input, one center is deleted.
    - In the segment addition step, centers are inserted graphically by the user. These centers must be defined within the actual boundaries of the CFU visible in intensity information of $\mathbf{I}_{in}$. The centers are used as seed points, first, for fast marching method to detect segments as BLOBs. If there are more centers for a single BLOB, then these centers contained inside the BLOB are used as seed points for watershed segmentation.
    - New segments are added and false one removed to form the final segmented image $\mathbf{I}_s$.

19. Useful features like area, eccentricity, radius, mean intensity etc. are calculated from $\mathbf{I}_s$. The results for total count and size of segments are plotted according to the type of input data and the other features can be saved in a summary *csv/ txt* file.

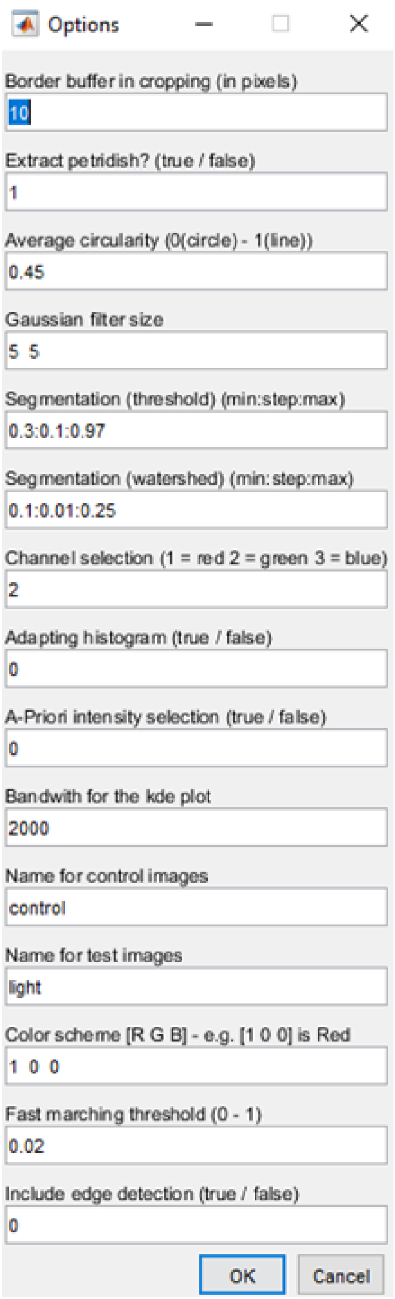

Figure 5. Options dialog of AutoCellSeg.

Supplementary data 3

The parameters and options (see Fig. 5) that the user can change in AutoCellSeg are:

1. The border buffer (in pixels) is used during cropping of the image within the internal process. For further segmentation of binary large objects (BLOBs) detected initially using thresholding, each detected BLOB should be cropped with a safe border. A value of 10 means that each BLOB is extracted with a border of 10 pixels on each side of the image.

2. Petri dish extractor enables the user to extract dish boundaries automatically if it is set to 1 (true). If the user does not wish to extract the petri dish, 0 or 'false' should be selected.

3. The average circularity represents the segments required for the feedback-based watershed operation. It ranges from 0

[4]This function can also be turned off manually if required.

(circle) to 1 (line) which by default is set to 0.45. This is different from the a priori circularity (eccentricity) extracted using GUI since it only guides watershed segmentation operation.

4. The Gaussian filter size is used to smooth the varying spatial image intensity. The size of filter is $s \times s$. The default value chosen is $s = 5$. For smaller images, having less spatial intensity variation, this value should be kept smaller.

5. Thresholding values vector. It must contain threshold values in the format (min:step:max). To make the program run faster, a vector of shorter length can be used. However, doing so may not ensure the optimal results.

6. Vector containing the parameter values for watershed in the format (min:step:max). Choosing very large max values may not be useful. The step should be much smaller than the step in the aforementioned vector for thresholding.

7. Channel selection (1 = red, 2 = green, 3 = blue). If 0 is chosen, then the resulting image contains the mean of all three channels at corresponding pixel locations.

8. The intensity histogram adaptation step. Based on the type of data information, a user can choose whether he wishes to adapt the intensity histogram equalization for the contrast enhancement (true / false).

9. Whether or not (true/ false) the intensity values should be included in the a-priori selection.

10. The bandwidth for the Kernel Density Estimation (KDE) function plot.

11. Name for control images.

12. Name for test images as per instruction given at the beginning of (Supplementary data 2).

13. The color scheme for delineation of segmentation as $[R,G,B]$ triplet between 0 (min) - 1 (max) e.g. $[0,0,1]$ means that boundaries of segments would be shown in blue color.

14. Threshold for fast marching method. It is a non-negative value between 0 and 1 and it specifies the level at which the fast marching method should threshold to produce a binary image.

15. Include edge detection (true/ false) uses Laplacian of Gaussian method to find edges. The threshold for edge detection is calculated automatically (using zero-crossings).

Supplementary data 4

A video demonstration on how to use the software in fully automated mode with correction is also provided. It includes data selection, selection of a priori information about the segments to be found, correction of the segmentation results, creation of plots, saving results and visualizing the results is also attached. The video file[5] is named as 'Supplementary video 1'.

Supplementary data 5

Ilastik+CellProfiler on new benchmark

The results from Ilastik+CellProfiler presented in 'Results' section of main manuscript are based on batch processing. This essentially meant that we labeled one image from each bacterial species for pixel classification in Ilastik. Since the results were not improved remarkably, we labeled each image across the whole benchmark individually. This requires some time/iterations to adequately label the input pixels for background and the colonies such that the resulting segmentation is close to the ground truth. Consequently, the results can be improved indicated by quality measures i.e. $Q_m = 0.95$ and $q_{1,m} = 5.4$. Some of the individual results were better than that of AutoCellSeg. However, it is not trivial in Ilastik+CellProfiler to do the post-editing for the correction of results. The resulting segmentation masks and the CellProfiler pipeline used for post-processing for each bacterial species can be found in its respective data folder at Github e.g. for E.coli [6]

[5] https://youtu.be/vwH-kO4hNSw
[6] https://github.com/AngeloTorelli/AutoCellSeg/tree/master/DATA/Benchmark/E.coli/Ilastik_CellProfiler

Batch processing

One data set with 49 images with a resolution of 2592 × 1944 pixels available on the OpenCFU project website[7] was used. The data set contains images of fluorescent *E.coli* colonies on agar plate expressing RFP and GFP using two different colors. This data set was chosen to evaluate the performance of AutoCellSeg in batch processing mode (fully automated option). Moreover, the results were compared with OpenCFU for the detection of the colonies (see Fig. 6). The additional advantage in AutoCellSeg is that after running the process in fully automated mode, one can add undetected colonies and remove the incorrectly detected colonies. The results obtained could only be compared to each other since there was no ground truth available at the aforementioned link from where the data was downloaded. The aim here is to show that with AutoCellSeg, a comparable count to OpenCFU can be obtained. This shows the versatility and applicability of AutoCellSeg on different data sets. Moreover, a user can easily discern the differences in colony boundaries detected.

OpenCFU was run with auto threshold mode selecting a minimum radius of 4 (global parameter setting). For AutoCellSeg the default parameters were used. First, both programs were used in fully automated mode without any post-processing and then manual correction was performed for both. This was done to see the room of improvement each software allows the user to correct the results.

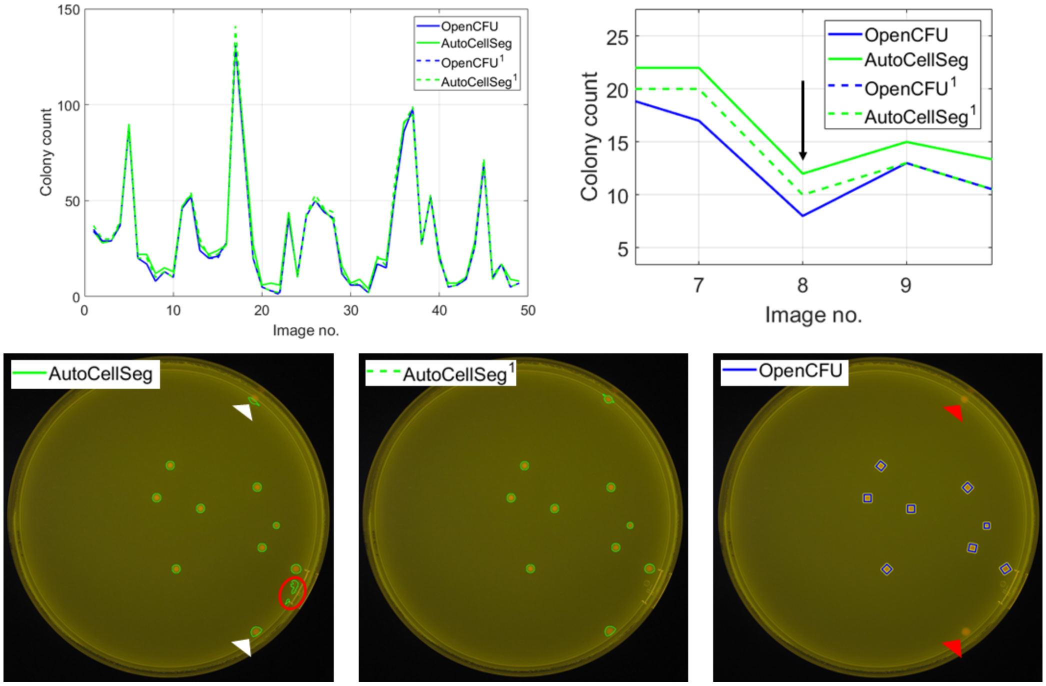

Figure 6. The plot on top-left shows the comparison of colony count between AutoCellSeg (green) and OpenCFU (blue) at each individual image. The plot on top-right is a zoomed-in window of the plot on top-left. Image number 8 of the data set is considered as an example and corresponding segmentation results from both software are shown in the images at the bottom. The first image from bottom shows the AutoCellSeg result in fully-automated mode without any correction. The image in the middle at the bottom shows the segmentation outcome using correction after the results were obtained from fully automated mode. The image on the bottom right shows the segmentation result from OpenCFU. It demonstrates the fully automated operation mode (solid lines) in comparison with adding the post-processing step (dashed lines) for the two software. The *subscript*^{1} refers to the addition of post-processing step after the results are obtained using the fully automated mode. The white arrowhead shows the detection of additional true positives that were missing in OpenCFU segmentation as indicated by red arrowheads. The red encirclement shows the detection of false positives. Note: OpenCFU cannot add new segments during correction step where as no false positive was detected to be removed. Therefore, the segmentation result does not require correction.

[7]https://sourceforge.net/projects/opencfu/files/samples/EcoliRobEgbert.zip/download

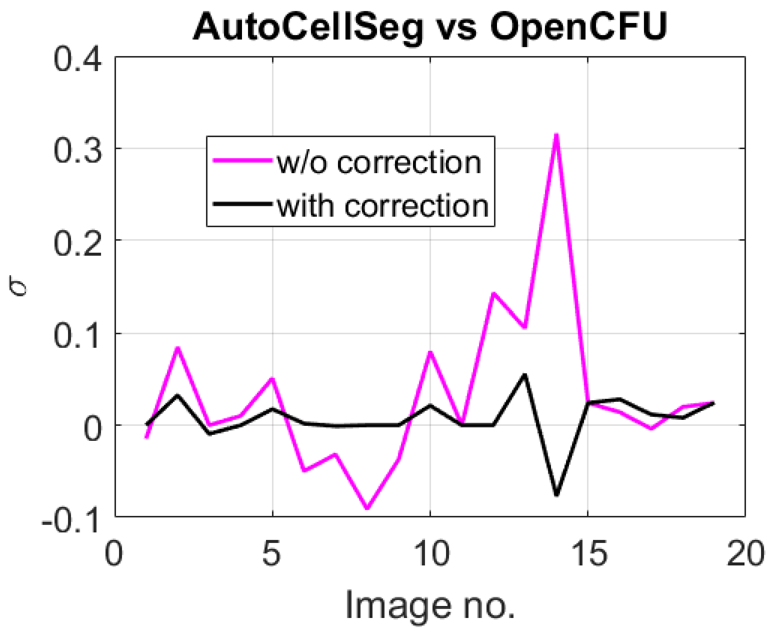

Figure 7. Measure of similarity between AutoCellSeg and OpenCFU based on the difference in colony count. The image number is given on x-axis. On y-axis, normalized difference in colony count σ according to criterion (8) is used in both modes (i.e. without correction (trend in magenta) and with correction (trend in black)).

Inverted background data evaluation

A normalized difference in colony count σ was used according to:

$$\sigma = \frac{n_{acs} - n_{ocfu}}{\max(n_{acs}, n_{ocfu})} \tag{8}$$

where, n_{acs} and n_{ocfu} are the number of colonies detected by AutoCellSeg and OpenCFU respectively. The negative values in graph shows the underestimation of colony count by AutoCellSeg with respect to count delivered by OpenCFU and positive values show overestimation. The correction step reduces the differences between the two tools used. The overall results for whole data set is shown in graph in Fig. 7. AutoCellSeg may underestimate/overestimate the number of colonies to a smaller extent, however, it has the advantage to add undetected additional colonies afterwards.

Cell segmentation

The segmentation of data sets based on human cells presents a different kind of challenge. The aim to use such data sets is to demonstrate that AutoCellSeg also performs well on cell-based images. Among existing data sets with ground truth, two data sets from the Broad Bioimage Benchmark Collection from the Broad Institute were used[8]. The first one is referenced as *BBBC007* which contains drosophila Kc167 cell images and the respective ground truth images as outlines[2,3]. From this data set, actin images for the evaluation were excluded and only the DNA images were kept. The ground truth images provided contains black outlines to represent cell boundaries where as the cells and background are white. The background was removed[9] to create the masks. The thickness of the outlines of more than one pixel was not taken into account and no other changes were made to the ground truth images. The second data set referenced as *BBBC008*[3] containing human HT29 colon-cancer cells images and the respective ground truth images as binary masks was used (see Fig. 8). For this data set, only the images of the first channel (DNA) were used for the comparison between AutoCellSeg and OpenCFU while excluding the third channel which stains actin in the cytoplasm.

For comparison, all results were converted to the same format as the ground truth masks. To have the same number of cells found by OpenCFU, cells with overlapping or touching boundaries are separated with the watershed method.

The segmentation quality of OpenCFU and AutoCellSeg with the ground truth provided was evaluated using nine different evaluation measures were used. Apart from Q, μ_1 and μ_2, other metrics were chosen from the five metrics previously used for a

[8]From https://data.broadinstitute.org/bbbc/image_sets.html

[9]By using the built-in MATLAB function *imclearborder*.

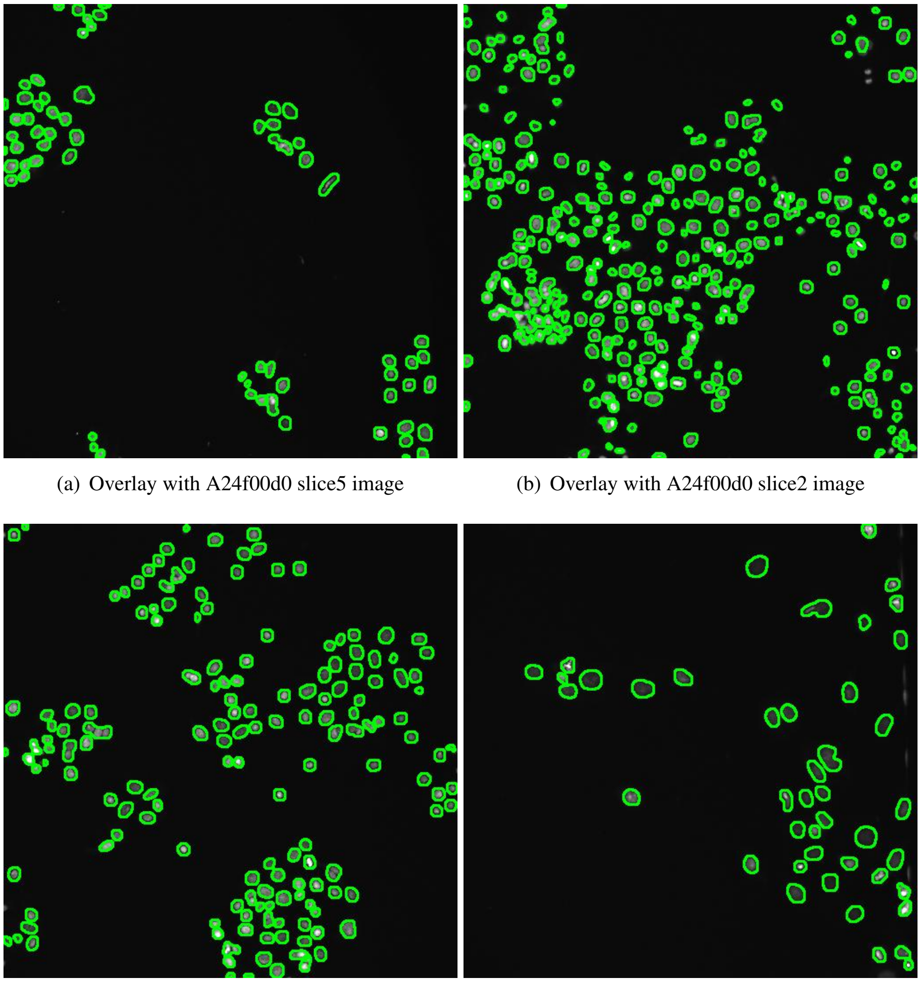

(a) Overlay with A24f00d0 slice5 image (b) Overlay with A24f00d0 slice2 image

(c) Overlay with A24f00d0 slice1 image (d) Overlay with L15f00d0 slice6 image

Figure 8. Randomly picked images with the respective borders found by AutoCellSeg.

ISBI Challenge[10]. These metrics are:

- local pixel-wise boundary prediction error (pixel error e_p),
- minimum mean square error between the pixels of the target segmentation and the pixels of a topology-preserving warped source segmentation called warping error (e_w)[4], and
- frequency with which two masks disagree over whether a pair of pixels belongs to the same or different objects, called rand error (e_r), which is closely related to rand index[5].

For the sake of completeness, we kept these established evaluation measurements[11] even though they come with some flaws i.e. pixel error considers only whether or not a given pixel was correctly classified as a boundary pixel, without concerning the ultimate effect of that prediction on the resulting image segmentation[6]. We also used[12] the foreground-restricted rand scoring (V^R) and information theoretic scoring (V^I)[6] which were introduced to compensate these flaws and to compare the results better in the presence of challenges. OpenCFU was run with auto threshold mode selecting a minimum radius of 10 (global parameter setting).

Both tools were used in automated mode with the manual correction. The results are shown in Tab. 1, where it can be seen that AutoCellSeg has a higher score and lower error than OpenCFU in almost all metrics. Only the warping error from the

[10]Segmentation of neuronal structures in EM stacks: http://brainiac2.mit.edu/isbi_challenge/
[11]http://imagej.net/Segmentation_evaluation_metrics_-_Script
[12]http://imagej.net/Segmentation_evaluation_after_border_thinning_-_Script

masks created from OpenCFU were better because the perfect circles affect this measure to an extremely lesser extent. From the results, it can be seen that measure μ_2 is somewhat more strict than e_p since it takes into account each individual pixel of each object instead of the entire image. For a comparison, the results of new benchmark data set was also added.

Data set	Tool	Q	μ_1	μ_2	V^R	V^I	e_p	e_w^*	e_r
**BBBC007**	1	**0.49**	**0.98**	**0.50**	**0.97**	**0.99**	**0.33**	2.2	**0.06**
	2	0.10	0.64	0.15	0.68	0.90	0.45	**0.6**	0.55
**BBBC008**	1	**0.72**	**0.99**	**0.73**	**0.93**	**0.98**	**0.21**	2.5	**0.13**
	2	0.21	0.57	0.35	0.62	0.89	0.42	**0.6**	0.57
**New**	1	**0.96**	**0.99**	**0.97**	**0.97**	**0.99**	**0.06**	0.4	**0.11**
	2	0.63	0.79	0.76	0.74	0.91	0.21	**0.2**	0.28

Table 1. Comparison between AutoCellSeg (tool 1) and OpenCFU (tool 2) using two different benchmark data sets i.e. BBBC007 and BBBC008 from the Broad Institute, and the new benchmark data set we created. The evaluation measures are: total quality (Q), count (μ_1), overlapping pixels (μ_2), foreground-restricted rand scoring (V^R), information theoretic scoring (V^I), local pixel-wise boundary prediction error (e_p), minimum mean square error between the pixels of the target segmentation and the pixels of a topology-preserving warped source segmentation (e_w^* unit in 10^{-4}) and frequency with which two masks disagree over whether a pair of pixels belongs to the same or different objects (e_r). The better results between the two software are highlighted in bold.

Single images - different colony types

Single images from different published experiments were taken to see the performance of AutoCellSeg on random images. A low resolution image of bacterial colony and an image of clonogenic colonies (right image in Fig. 9) provided by Choundhry et al.[7] was segmented with AutoCellSeg. Choundhry et al.[7] also provides the manual count of the colonies for a comparison which are shown in white on the top-left corner of the images of Fig. 9.

The result for the low resolution image is shown in the top-left image in Fig. 9. The count from AutoCellSeg is quite close to the count provided ($n = 23$). The CFUs in the image with clonogenic colonies are blue colored on a light background and vary in size to a larger extent. The segmentation result for this image is shown in the big image on the right side of Fig. 9. The count from AutoCellSeg also matches the count provided ($n = 51$).

The last image (bottom-left image in Fig. 9) was taken randomly from the data set provided by Ferrari et al.[8] and compared with the provided label. The images from this data set were categorized into aggregates containing one to six colonies of bacteria on blood agar plates. AutoCellSeg successfully segmented the five colonies at the center which corresponds to the given mask that was classified as a five colony aggregate according to the ground truth.

Parameter adjustments

In case of batch data processing, the following settings were changed from the default values for the AutoCellSeg operation:

```
Gaussian filter size:1 1
Segmentation(threshold)(min:step:max):.3:.2:.97
Segmentation(watershed)(min:step:max):.01:.03:.3
```

For inverted background application, following parameters were changed from default for AutoCellSeg:

```
Extract petridish?(1=true/0=false):0
Gaussian filter size:3 3
Segmentation(threshold)(min:step:max):.3:.3:.97
Segmentation(watershed)(min:step:max):.1:.01:.25
Include edge detection(1=true/0=false):1
```

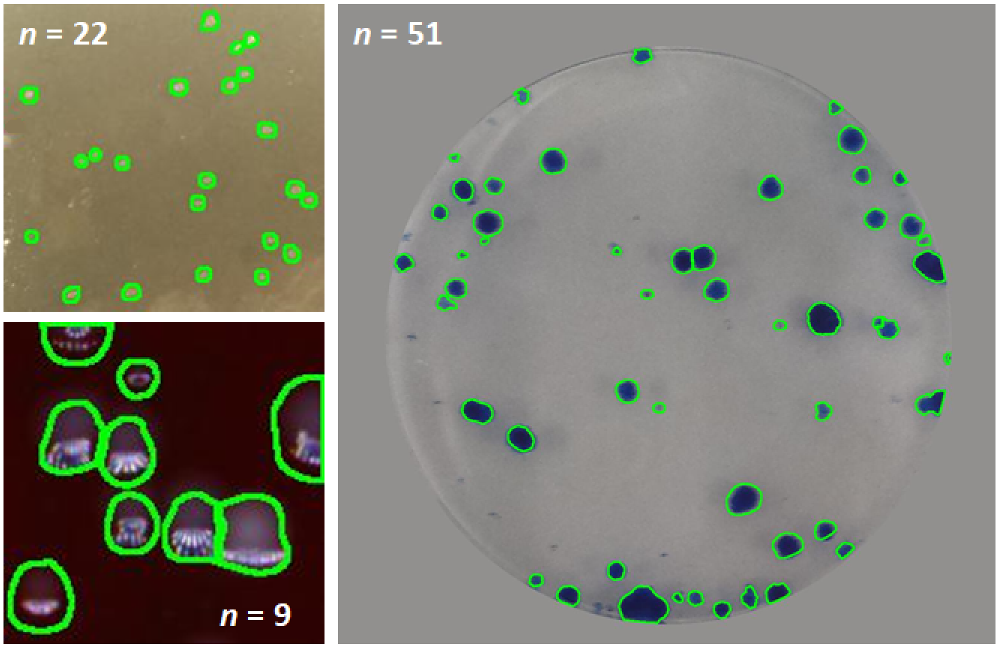

Figure 9. Segmentation of diverse images by AutoCellSeg using partially automated mode using correction. The image at top left and the image on right are taken from Choundhry et al.[7] and the image at the bottom on left was chosen randomly from Ferrari et al.[8]. The colony count for each image is given on the corners of images.

Comparison overview of the tools used

Features\Programs	AutoCellSeg	OpenCFU	CellProfiler	Ilastik+ (batch)	Ilastik+ (ind)
**Execution speed**	✓✓	✓✓✓	✓	✓	✓
**Accuracy**	✓✓✓	✓✓	✓✓	✓✓	✓✓✓
**Degree of automation**	✓✓✓	✓✓	✓✓	✓	✓
**Data analysis**	✓✓✓	✓	✓✓	✓✓	✓✓
**All-in-one solution**	✓✓✓	✓✓	✓✓	✓	✓
**User-friendly**	✓✓✓	✓✓	✓	✓	✓
**A-priori labeling**	✓✓✓	✓	✓✓	✓✓✓	✓✓✓
**Segment correction**	✓✓✓	✓✓	✓	✓	✓

Table 2. Performance and features comparison between the used frameworks. ✓is the minimum or baseline among these frameworks. ✓✓shows an improvement in compared to the baseline and ✓✓✓represents the best option amongst the tools. 'Ilastik+' refers to the combination of Ilastik+CellProfiler and 'ind' refers to the complete labeling of the whole benchmark using each individual image.

References

1. Sethian, J. A. A fast marching level set method for monotonically advancing fronts. *Proc. Natl. Acad. Sci.* **93**, 1591–1595 (1996).

2. Jones, T., Carpenter, A. & Golland, P. Voronoi-based segmentation of cells on image manifolds. *Comput. Vis. for Biomed. Image Appl.* 535–543 (2005).

3. Ljosa, V., Sokolnicki, K. L. & Carpenter, A. E. Annotated high-throughput microscopy image sets for validation. *Nat. methods* **9**, 637–637 (2012).

4. Jain, V. *et al.* Boundary learning by optimization with topological constraints. In *Computer Vision and Pattern Recognition (CVPR), 2010 IEEE Conference on*, 2488–2495 (IEEE, 2010).

5. Rand, W. M. Objective criteria for the evaluation of clustering methods. *J. Am. Stat. association* **66**, 846–850 (1971).

6. Arganda-Carreras, I. *et al.* Crowdsourcing the creation of image segmentation algorithms for connectomics. *Front. neuroanatomy* **9** (2015).

7. Choudhry, P. High-throughput method for automated colony and cell counting by digital image analysis based on edge detection. *PloS one* **11**, e0148469 (2016).

8. Ferrari, A., Lombardi, S. & Signoroni, A. Bacterial colony counting with convolutional neural networks in digital microbiology imaging. *Pattern Recognit.* **61**, 629–640 (2017).
